# Supplementary material for: Initial Amino Acid:Codon Assignments and Strength of Codon:Anticodon Binding
Source: J Am Chem Soc. 2024 Apr 27;146(18):12857–63. doi: 10.1021/jacs.4c03644 (PMC11082893; doi:10.1021/jacs.4c03644)
Supplement: Supplementary file 1 — ja4c03644_si_001.pdf [file ja4c03644_si_001.pdf]

Supporting Information

Initial amino acid:codon assignments and strength of codon:anticodon binding

Meng Su, Samuel J. Roberts, John D. Sutherland*

MRC Laboratory of Molecular Biology, Francis Crick Avenue, Cambridge Biomedical Campus, Cambridge CB2 0QH, United Kingdom.

*Correspondence to: johns@mrc-lmb.cam.ac.uk

This PDF file includes:

Figures S1 to S35

Tables S1 to S12

Further Details of the Deacylation Hypothesis

Transpeptidation between two free aminoacyl-tRNAs apparently creates one free tRNA and one dipeptidyl-RNA – as the free aminoacyl-tRNAs are more hydrolytically unstable than dipeptidyl-tRNAs, transpeptidation appears to be reducing the number of free tRNAs available after limited hydrolysis rather than increasing them. Firstly, other things being equal, transpeptidation between a free aminoacyl-tRNA and an acyl-aminoacyl-tRNA produces a free tRNA and an acyl-dipeptidyl-RNA – after hydrolysis, both the pre-transpeptidation state and the post-transpeptidation state lead to one free and one blocked tRNA. However, other things are not equal, in fact they are decidedly unequal – if the free aminoacyl-tRNA is a serinyl-tRNA, literature data suggests that the hydroxymethyl group of this species should be about a 50-100 times better nucleophile when it comes to attacking the ester group of an (acyl-)aminoacyl-tRNA than the amino group of any aminoacyl-tRNA.[1] Acylation of the serinyl-hydroxymethyl group is followed by *O*- to *N*- migration leaving the hydroxymethyl group to again function as a de(acyl)-aminoacylating nucleophile again. Thus, using serinyl-tRNA as the nucleophilic component leads to a net increase of free tRNAs. The ribosome might have been 'trained' on this easier chemistry preconfiguring it to be able to function using direct N-nucleophilic chemistry at a later point.

1. Weber, A. L.; Orgel, L. E. The formation of peptides from the 2′(3′)-glycyl ester of a nucleotide. *J. Mol. Evol.,* **1978**, *11* (3), 189–198. 10.1007/BF01734480.

Discussion on exceptions involving family-box codons with less optimal binding affinity

We observed that 12 out of 32 family-box codons exhibited K_D values larger than 130 μM. These specific codons are highlighted in red in the table below.

BLI	U	C	A	G	
U	/	260	/	/	U
	450	75	1500	/	C
	/	280	/	/	A
	240	310	210	370	G
C	150	230	510	75	U
	65	14	230	90	C
	/	75	/	36	A
	730	75	270	85	G[a]
A[d]	/	530	/	170	U[d]
	1000	27	860	180	C
	420	150	/	1000	A
	330	130	1400	85	G
G	470	120	190	115	U
	35	17	230	60	C[a]
	400	95	780	130	A
	160	38	140	35	G

We attribute the suboptimal binding affinities to two factors:

1) The solubility of the anticodon loops is compromised, particularly in cases where loops contain multiple purines. These loops, which pair with codons UCU, CUU, CCU, ACU, and GUU, are less soluble in the aqueous buffer.

2) Certain loops have a propensity to form self-dimers in our model, thereby reducing the availability of free anticodon loops in solution (Fig. S15). Consequently, this leads to an apparent decrease in binding affinity, as observed with loops pairing to UCA, CUG, and ACU.

The K_D of the codon AGG binding to its corresponding loops exhibits a low value of 85 μM (highlighted in blue), equivalent to the K_D of CGG. We maintain that even though AGG may form stable sequential bindings on a ssRNA, it does not alter our hypothesis that "family-box codons bind tighter." Arginine is designated to AGG/AGA and CGN boxes, and all CGN codons display strong binding to their anticodon loops. Consequently, arginine can undergo transpeptidation with both CGN and AGG codon-anticodon loop interactions.

Materials and Methods

Materials and general methods

Reagents and solvents were obtained from *Acros Organics*, *Santa Cruz Biotechnology*, *Sigma-Aldrich*, and were used without further purification. pH was measured using a *Mettler Toledo* SevenEasy pH Meter S20 combined with a *ThermoFisher Scientific* Orion 8103BN Ross semi-micro pH electrode or an Orion™ 9810BN Micro pH Electrode. ^{1}H-, and ^{13}C-nuclear magnetic resonance (NMR) spectra were acquired using a *Bruker* Ultrashield 400 Plus or *Bruker* Ascend 400 operating at 400.13, and 100.61 MHz, respectively. Data for ^{1}H- experiments are reported as chemical shifts (relative integral, splitting, coupling constant, assignment) whilst those for ^{13}C- are reported as chemical shifts (assignment). Chemical shifts (δ) are shown in ppm. Coupling constants (J) are given in Hertz and the notations s, d, and t represent the multiplicities singlet, doublet and triplet, respectively. Yields from NMR were calculated by integrating the most distinct peak of each species and dividing by the total normalised integral of each species. Species identities were confirmed by spiking with solutions of authentic samples of known quantities at the same pH as the bulk sample. The increase in integral was then cross-referenced against the known amount added to provide a further method of yield quantification. Analytical High-Pressure Liquid Chromatography (HPLC) was run on Dionex Ultimate 3000 (*Thermo Scientific*) using an Atlantis T3, 3 µm, 4.6 × 250 mm column. Preparative High-Pressure Liquid Chromatography (HPLC) was run on a *Varian Prep Star* preparative HPLC system with a *Varian Pro Star* UV/vis detector and a *Water Atlantis* T3 Prep OBD 5 µM 19 × 250 mm column.

Peptide bond formation from glycyl-adenosine-5'-*O*-methylphosphate (415 mM)

Glycyl-adenosine-5'-*O*-methylphosphate (40 µL, 800 mM in water) was diluted with water (20 µL) and the pH was raised to 8.2 with NaOH (5 M, 17 µL), making a solution with a final adenylate concentration of 415 mM. The solution was kept at room temperature whilst the pH was monitored and maintained at 8.2 over 2.5 hours (as the Orgel results suggested yields plateaued within 2 hours), after which the pH was raised to 12.5 with NaOH (5 M, 17 µL) for 5 minutes at room temperature. The pH was reduced to 10 with HCl (2 M). The final concentration of adenosine-5'-*O*-methylphosphate was measured by nanodrop UV/Vis spectrometry to be 273 mM (assuming an extinction coefficient of 15000 M^{-1} cm^{-1}). An aliquot (20 µL) was removed for functionalisation by dansyl chloride, and the remaining solution was diluted with D_2O (400 µL) and analysed by NMR and dansyl labelling (Fig. S1, Fig. S3B).

Peptide bonds formation from glycyl-adenosine-5'-*O*-methylphosphate (35 mM)

Glycyl-adenosine-5'-*O*-methylphosphate (4 µL, 800 mM in water) was diluted with water (56 µL) and the pH was raised to 8.2 with NaOH (1 M, 32 µL), making a solution of adenylate with a final concentration of 35 mM. The solution was kept at room temperature whilst the pH was monitored and maintained at 8.2 over 2.5 hours, after which the pH was raised to 12.5 with NaOH (5 M, 17 µL) for 5 minutes at room temperature. The pH was reduced to 10 with HCl (2 M). The final concentration of adenosine-5'-*O*-methylphosphate was measured by nanodrop UV/vis spectrometry to be 29 mM (assuming an extinction coefficient of 15000 M^{-1} cm^{-1}). A 20 µL aliquot was removed for functionalisation

by dansyl chloride, and the remaining solution was diluted with D_2O (400 µL) and analysed by NMR and dansyl labelling (Fig. S2, Fig. S3C).

General method for the dansylation of crude mixtures

An aliquot (1 µL) of crude products of peptide formation was diluted to 0.1 M. This solution (1 µL) was combined with a solution of benzylamine (1 µL, 0.1 M), then added to $NaHCO_3$ solution (40 mM, pH 9.4, 398 µL). Dansyl chloride in acetonitrile (5 mM, 200 µL) was added and the solution was kept on ice and maintained at pH 9.2 with NaOH solution (1 M). Pyridine (4%, 20 µL) was added and the solution was analysed by analytical HPLC (Buffer A: 10 mM formic acid, Buffer B: acetonitrile; 15% B at 0 min, 15% B at 1 min, 50% B at 23 min, 95% B at 33.5 min).

Yields were calculated by the integration of peaks that had the same retention time as those of the authentic synthetic standards, followed by normalisation against the integration of the *N*-dansyl-benzyl amine. This method was the product of extensive optimisation by comparison of the yields of dansylation of a mixture of glycine, diglycine and triglycine at a total concentration of 0.1 M with the integrals seen in the ^{1}H NMR spectra of the same mixture of glycines (Fig. S3, Table S1).

Peptide bonds from glycyl-adenosine-5'-*O*-methylphosphate and free glycine (both 207 mM)

Glycyl-adenosine-5'-*O*-methylphosphate (20 µL, 800 mM in water) was diluted with water (33 µL) and combined with a solution of glycine (20 µL, 800 mM in water). The pH was raised to 8.2 with NaOH (5 M, 4 µL), making a solution with a final concentration of glycine species of 415 mM. The solution was kept at room temperature whilst the pH was monitored and maintained at 8.2 over 2.5 hours, after which the pH was raised to 12.5 with NaOH (5 M, 17 µL) for 5 minutes at room temperature. The pH was reduced to 10 with HCl (2 M). The final concentration of adenosine-5'-*O*-methylphosphate was measured by nanodrop UV/vis spectrometry to be 168 mM (assuming an extinction coefficient of 15000 M^{-1} cm^{-1}). A 20 µL aliquot was removed for functionalisation by dansyl chloride, and the remaining solution was diluted with D_2O (400 µL) and analysed by NMR and dansyl labelling (Fig. S4, Fig. S3D).

Di-glycyl-adenosine-5'-*O*-methylphosphate hydrolysis

Di-glycyl-adenosine-5'-*O*-methylphosphate (8 µL, 553 mM in water) was diluted with D_2O (432 µL) the pD was raised to 8.2 with NaOD (1 M), making a solution of final concentration 10 mM. The solution was kept at room temperature whilst pH was monitored and maintained at 8.2 for 16 hours. Over this period the solution was periodically analysed by NMR (Fig. S5, S6). The calculated half-life from manual fitting ($Yield = Yield_0\, e^{-0.012t}$) was 58 minutes. DKP and diglycyl adenosine were identified, as products in a 3:1 ratio.

Peptide bond formation from formyl-glycyl- and glycyl-adenosine-5'-*O*-methylphosphate (207 mM both)

Glycyl-adenosine-5'-*O*-methylphosphate (20 µL, 800 mM in water) was diluted with water (16.7 µL) and combined with a solution of *N*-formyl-glycyl-adenosine-5'-*O*-methylphosphate (32.3 µL, 496 mM in water). The pH was raised to 8.2 with NaOH (5 M, 8 µL), making a solution of a final concentration of 415 mM. The solution was kept at room temperature whilst the pH was monitored and maintained at 8.2 over 2.5 hours, after which the pH was raised to 12.5 with NaOH (5 M, 17 µL) for 5 minutes at room temperature. The pH was reduced to 10 with HCl (2 M). The final concentration of adenosine-5'-*O*-methylphosphate was measured by nanodrop UV/vis spectrometry to be 227 mM (assuming an extinction coefficient of 15000 M^{-1} cm^{-1}). An aliquot (20 µL) was removed for functionalisation by dansyl chloride, and the remaining solution was diluted with D_2O (400 µL) and analysed by NMR and dansyl labelling (Fig. S7, Fig. S3E).

Synthesis

Adenosine-5'-*O*-methylphosphate

Adenosine-5'-*O*-monophosphate (acid form, 3.0 g, 8.64 mmol), *N,N*-di-isopropyl-ethyl amine (3.0 mL, 17.28 mmol) and DCC (9.0 g, 43.3 mmol) were dissolved in methanol (90 mL) and stirred at room temperature for 64 hours. NaOH solution (1 M, 18.9 mL) was added and then the combined solution was evaporated to dryness *in vacuo*. The solids were resuspended in water (90 mL), filtered and the solution was lyophilised. The powder was resuspended in water (90 mL), filtered and lyophilised affording adenosine-5'-(*O*-methylphosphate) 3.30 g as a white powder (quantitative yield).

^{1}H NMR (400 MHz, D_2O) δ = 8.37 (1H, s, H8), 8.18 (1H, s, H2), 6.04 (1H, d, *J* = 5.6 Hz, H1'), 4.41 (1H, dd, J = 5.6, 3.3 Hz, H3'), 4.29 (1H, p, J = 3.3 Hz, H4'), 4.03 (2H, m, H5'), 3.44 (3H d, J = 10.8 Hz, Me-H).

^{13}C NMR (100 MHz, D_2O) δ = 155.6 (C6), 152.8 (C2), 149.1 (C4), 118.5 (C5), 139.7 (C8), 87.8 (C1'), 83.9 (C4'), 74.9 (C2'), 70.9 (C3'), 65.1 (C5'), 52.8 (Me).

^{31}P NMR (162 MHz, D_2O) δ = 1.58.

Mass: 360.07 [M-1] calculated, 359.9 observed.

Glycyl-adenosine-5'-*O*-methylphosphate

N-Boc-glycine (175 mg, 1.00 mmol) and carbonyl diimidazole (161 mg, 1.00 mmol) were dissolved in acetonitrile (9.3 mL) and mixed for 10 minutes at room temperature. Adenosine-5'-(*O*-methylphosphate) (350 mg, 0.913 mmol) was dissolved in water (5.0 mL) and added to the acetonitrile mix. The reaction was stirred at room temperature for 10 minutes and then adjusted to pH 4 with HCl (2 M). The solution was reduced to dryness *in vacuo* and then purified by preparative HPLC using (Buffer A: 20 mM formic acid, Buffer B: acetonitrile, 0-20% over 45 minutes). Fractions containing the product were collected and lyophilized. The resultant powder was redissolved in trifluoroacetic acid (2.5 mL) and stirred for 20

minutes at room temperature before concentrating *in vacuo*. The products were purified by preparative HPLC using (Buffer A: 20 mM formic acid, Buffer B: acetonitrile, 0-20% over 45 minutes). Fractions containing the product were collected and lyophilized. Diethyl ether (10 mL × 3) was repeatedly added to the lyophilizate. The resultant suspension was sonicated and then the ether evaporated *in vacuo*. The resulting clear oil was dissolved in 150 µL water and the concentration was measured by nanodrop UV/Vis spectroscopy. Assuming an extinction coefficient of 15000 M^{-1} cm^{-1} the concentration was measured to be 1.08 M (17% yield). a = 2' acylated, b = 3' acylated.

^{1}H NMR (400 MHz, d_6-DMSO) δ = 8.57(1H, s, H8b), 8.53 (0.3H, s, H8a), 8.44 (2.6H, br. s, NH_2), 8.35 (1.3H, s, H2b), 6.95 (3.9H, brs, NH_3^+), 6.27 (0.3H, d, J = 4.8 Hz, H1'a), 6.04 (1H, d, J = 6.7 Hz, H1'b), 5.81 (0.3H, t, J = 4.8 Hz, H2'a), 5.48 (1H, dd, J = 5.2, 2.4 Hz, H3'b), 4.96 (1H, dd, J = 6.7, 5.2 Hz, H2'b), 4.60 (0.3H, t, J = 4.8 Hz, H3'a), 4.37 (1H, m, H4'b), 4.25-4.00 (2.9H, m, H4'a + H5'a + H5'b), 3.97 (2H, d, J = 4.6, αb), 3.91 (0.6H m, αa), 3.52 (3H d, J = 10.5 Hz, Me-H), 3.50 (0.9H d, J = 10.5 Hz, Me-H).

^{13}C NMR (100 MHz, d_6-DMSO) δ = 166.9 (ester), 153.9 (C6), 149.9 + 149.6 + 149.3 + 148.9 (C2 + C4), 140.6 + 140.3 (C8), 118.9 (C5), 86.7 (C1'b), 85.0 (C1'a), 83.3 (C4'a), 81.1 (C4'b), 76.3 (C2'a), 74.5 (C3'b), 72.2 (C2'b), 68.9 (C3'a), 65.3 (C5'), 53.1 (Me), 40.1 (α).

^{31}P NMR (162 MHz, d_6-DMSO) δ = -0.37.

Mass: 417.09 [M-1] calculated, 416.9 observed.

Diglycyl-adenosine-5'-*O*-methylphosphate

N-Boc-diglycine (107 mg, 0.46 mmol) and carbonyl diimidazole (74 mg, 0.46 mmol) were dissolved in acetonitrile (4.6 mL) and mixed for 10 minutes at room temperature. Adenosine-5'-*O*-methylphosphate (80.5 mg, 0.42 mmol) was dissolved in water (2.5 mL) and added to the acetonitrile mix. The reaction was stirred at room temperature for 10 minutes and then adjusted to pH 4 with HCl (2 M). The solution was reduced to dryness *in vacuo* and then purified by preparative HPLC. Fractions containing the product were collected and lyophilized. The resultant powder was redissolved in trifluoroacetic acid (2.50 mL) and stirred for 20 minutes at room temperature before concentrating *in vacuo*. The products were purified by preparative HPLC. Fractions containing the product were collected and lyophilized. Diethyl ether (3 × 10 mL) was repeatedly added to the lyophilizate. The resultant suspension was sonicated and then the ether evaporated *in vacuo*. The resulting clear oil was dissolved in 56 µL water and the concentration was measured by nanodrop UV/Vis spectroscopy. Assuming an extinction coefficient of 15000 M^{-1} cm^{-1} the concentration was measured to be 553 mM (7% yield). a = 2' acylated, b = 3' acylated.

^{1}H NMR (400 MHz, d_6-DMSO) δ = 8.91 (1H, t, J = 5.4, amide-Hb), 8.80 (0.3H, t, J = 6.3, amide-Ha), 8.71 (0.3H, t, J = 5.5, DKP-amide), 8.46 (1H, s, H8b), 8.42 (0.3H, s, H8a), 8.25 (1H, s, H2b), 8.24 (0.3H, s, H2a), 8.14 (3.9H, m, NH_3^+), 7.81 (2H, br. s, NH_2b), 7.76 (0.6H, br. s, NH_2a), 6.21 (0.3H, d, J = 4.8 Hz, H1'a), 5.98 (1H, d, J = 6.7 Hz, H1'b), 5.74 (0.3H, t, J = 4.8 Hz, H2'a), 5.37 (1H, dd, J = 5.3, 2.9 Hz, H3'b), 4.93 (1H, dd, J = 6.7, 5.3 Hz, H2'b), 4.56 (0.3H, t, J = 4.8 Hz, H3'a), 4.32 (1H, m, H4'b), 4.18-4.00 (5.2H, m, H4'a, H5'a, H5'b, internal-αb, DKP-α), 3.87 (0.6H, d, J = 5.5, internal-αa), 3.67 (2H brs,

terminal- αb), 3.62 (0.6H brs, terminal-αa), 3.52 (3H d, J = 11.0 Hz, Me-H), 3.51 (0.9H d, J = 11.0Hz, Me-H).

^{13}C NMR (100 MHz, d_6-DMSO) δ = 171.2 (amide), 169.1, 167.0 (ester), 155.5 (C6), 151.8 (C2), 149.7 (C4), 140.2 (C8), 119.4 (C5), 87.3 (C1'b), 85.7 (C1'a), 83.4 (C4'a), 81.2 (C4'b), 75.9 (C2'a), 73.9 (C3'b), 72.2 (C2'b), 69.1 (C3'a), 65.5 (C5'), 53.3 (Me), 40.7, 40.1 (α).

^{31}P NMR (162 MHz, d_6-DMSO) δ = -0.44.

Mass: 474.09 [M-1] calculated, 473.9 observed.

***N*-Formyl-glycyl-adenosine-5'-*O*-methylphosphate**

N-Formyl-glycine (69.0 mg, 0.66 mmol) and carbonyl diimidazole (106 mg, 0.66 mmol) were dissolved in acetonitrile (6 mL) and mixed for 10 minutes at room temperature. Adenosine-5'-*O*-methylphosphate (210 mg, 0.548 mmol) was dissolved in water (3 mL) and added to the acetonitrile mix. The reaction was stirred at room temperature for 10 minutes and then adjusted to pH 4 with HCl (2 M). The solution was reduced to dryness *in vacuo* and then purified by preparative HPLC. Fractions containing the product were collected and lyophilized. Diethyl ether (3 × 10 mL) was repeatedly added to the lyophilizate. The resulting suspension was sonicated and then the ether evaporated *in vacuo*. The resulting clear oil was dissolved in 170 μL water and the concentration was measured by nanodrop UV/Vis spectroscopy. Assuming an extinction coefficient of 15000 M^{-1} cm^{-1} the concentration was measured to be 813 mM (24% yield). a = 3' acylated, b = 2' acylated.

^{1}H NMR (400 MHz, d_6-DMSO) δ = 8.62 + 8.54 (1H, t, J = 6.0, amide), 8.51 (1H, s, H8), 8.44 (1H, s, H8), 8.17 + 8.15 (2H + 2H, s, H2 + Formyl-H), 7.34 (2H, br. s, NH_2), 7.30 (2H, br. s, NH_2), 5.96 (1H, d, J = 7.1 Hz, H1'a), 5.93 (1H, d, J = 5.5 Hz, H1'b), 5.38 (1H, dd, J = 5.3, 1.5 Hz, H3'a), 4.95 (1H, dd, J = 7.1, 5.3 Hz, H2'a), 4.60 (1H, t, J = 5.5 Hz, H2'b), 4.23 (1H, m, H4'a), 4.19 (1H, t, J = 4.1 Hz, H3'b), 4.05 (4H, s, Hα + H4'b), 3.78-3.98 (6H, m, Hα + H5'a + H5'b), 3.37 (3H d, J = 10.3 Hz, Me-H), 3.35 (3H d, J = 10.6 Hz, Me-H).

^{13}C NMR (100 MHz, d_6-DMSO) δ = 169.3 (ester), 161.9 (amide), 156.4 (C6), 153.1 (C2), 150.2 (C4), 139.3 (C8), 119.3 (C5), 87.0 (C1'), 86.3 (C1'), 84.5 (C4'b), 81.6 (C4'a), 74.3 (C3'a), 73.9 (C2'b), 72.2 (C'2a), 70.9 (C3'b), 64.8 (C5'), 64.5 (C5'), 51.8 (Me), 39.5 (α).

^{31}P NMR (162 MHz, d_6-DMSO) δ = -0.3.

Mass: 445.1 [M-1] calculated, 444.9 observed.

***N*-Formyl-diglycine**

Diglycine (500 mg, 3.78 mmol) was dissolved in neat formic acid (8 mL) and heated to 55°C. Acetic anhydride (3.0 mL) was added dropwise over 5 minutes. The reaction was allowed to cool to room temperature whilst stirring for 1 hour. Ice was added and the sample was kept at 4°C overnight. The sample was then lyophilised affording 495 mg of product (82% yield).

^{1}H NMR (400 MHz, D_2O) δ = 8.15 (1H, s, formyl-H), 3.99 (2H, s, α), 3.93 (2H, s, α);

^{13}C NMR (100 MHz, D_2O) δ = 176.6, 170.9, 165.0, 43.4, 41.2.

Mass: 159.0 [M-1] calculated, 159.0 observed.

N-Formyl-triglycine

Triglycine (140 mg, 0.74 mmol) was dissolved in neat formic acid (2.0 mL) and heated to 55°C. Acetic anhydride (0.75 mL) was added dropwise over 5 minutes. The reaction was allowed to cool to room temperature whilst stirring for 1 hour. Ice was added and the sample was kept at 4°C overnight. The sample was then lyophilised affording 159 mg of product (quantitative yield).

^{1}H NMR (400 MHz, D_2O, pD 10.1) δ = 8.14 (1H, s, formyl-H), 3.99 (2H, s, α), 3.93 (2H, s, α), 3.71 (1H, s, α);

^{13}C NMR (100 MHz, D_2O) δ = 176.5, 171.8, 171.2, 165.1, 43.3, 42.5, 41.3.

Mass: 216.1 [M-1] calculated, 216.0 observed.

N-Dansyl-glycine

Glycine (30.1 mg, 0.408 mmol) in NaOH aq. (1 M, 0.5 mL) was added to dansyl chloride (100 mg, 0.371 mmol) in THF (3.5 mL) and stirred for 1 hour at room temperature. During that time the pH was monitored and kept above 9 with additional additions of 1 M NaOH. The solution was washed with ethyl acetate (3 × 10 mL). The aqueous layer was acidified to pH 4 with 1 M HCl and then washed with ethyl acetate (3 × 10 mL). The combined acidic organic layers were dried over anhydrous magnesium sulphate and then concentrated *in vacuo* affording 2 mg of *N*-dansyl-glycine (1.4% unoptimised yield – sufficient to provide an authentic product standard).

^{1}H NMR (400 MHz, d_6-DMSO) δ = 8.44 (1H, d, J = 8.8 Hz,), 8.29 (1H, d, J = 8.8 Hz), 8.10 (1H, d, J = 7.1), 7.58 (2H, m), 7.24 (1H, d, J = 7.5), 3.59 (2H, s, α), 2.82 (6H, s, Me).

^{13}C NMR (100 MHz, d_6-DMSO) δ = 170.3 (Carboxylic acid), 151.3 (N-Ar), 136.4, 129.3, 129.1, 127.8, 127.8, 123.5, 119.4, 115.1, 45.1 (Me), 43.8 (α).

Mass: 307.1 [M-1] calculated, 307.0 observed.

N-Dansyl-diglycine

Diglycine (53.9 mg, 0.408 mmol) in NaOH aq. (1 M, 0.5 mL) was added to dansyl chloride (100 mg, 0.371 mmol) in THF (3.5 mL) and stirred for 1 hour at room temperature. During that time the pH was monitored and kept above 9 with additional additions of 1 M NaOH. The solution was washed with ethyl acetate (3 × 10 mL). The aqueous layer was acidified to pH 4 with 1 M HCl and then washed with ethyl acetate (3 × 10 mL). The combined acidic organic layers were dried over anhydrous magnesium sulphate and then concentrated *in vacuo* affording 18.8 mg of *N*-dansyl-diglycine (13% yield).

^{1}H NMR (400 MHz, d_6-DMSO) δ = 8.46 (1H, d, J = 8.8 Hz), 8.29 (2H, d, J = 8.8 Hz), 8.09 (3H, m), 7.58 (2H, q, J = 8.0), 7.26 (1H, d, J = 7.8), 3.68 (2H, d, J = 5.6, 3.50α), (2H, s, α), 2.83 (6H, s, Me).

^{13}C NMR (100 MHz, d_6-DMSO) δ = 170.9 (carboxylic acid), 168.0 (amide), 151.2 (N-Ar), 135.9, 129.4, 129.0, 128.0, 127.9, 123.5, 119.2, 115.1, 45.1 (Me), 44.9 (α), 40.6 (terminal-α).

Mass: 364.1 [M-1] calculated, 364.0 observed.

N-Dansyl-triglycine

Triglycine (77.2 mg, 0.408 mmol) in NaOH aq. (1 M, 0.5 mL) was added to dansyl chloride (100 mg, 0.371 mmol) in THF (3.5 mL) and stirred for 1 hour at room temperature. During that time the pH was monitored and kept above 9 with additional additions of 1 M NaOH. The solution was washed with ethyl acetate (3 × 10 mL). The aqueous layer was acidified to pH 4 with 1 M HCl and then washed with ethyl acetate (3 × 10 mL). The combined acidic organic layers were dried over anhydrous magnesium sulphate and then concentrated *in vacuo* affording 18.4 mg of *N*-dansyl-triglycine (11% yield).

^{1}H NMR (400 MHz, d_6-DMSO) δ = 8.46 (1H, d, J = 8.4 Hz,), 8.29 (1H, d, J = 8.8 Hz), 8.11 (4H, m), 7.60 (2H, m), 7.26 (1H, d, J = 7.5), 3.74 (2H, d, J = 5.9, α), 3.66 (2H, d, J = 5.7, α), 3.50 (2H, s, α), 2.83 (6H, s, Me).

^{13}C NMR (100 MHz, d_6-DMSO) δ = 171.0, 168.8, 167.9 (amide, carboxylic acid), 151.5 (N-Ar), 136.4, 129.5, 129.1, 128.2, 127.9, 123.5, 119.2, 115.2, 45.1 (Me), 44.7 (α), 41.7 (α), 40.2 (α).

Mass: 421.1 [M-1] calculated, 421.0 observed.

Solid phase synthesis of RNA oligomers

All biotin-labelled RNA oligomers were purchased from *Sigma-Aldrich* and used without further purity checks. All other RNA oligomers were synthesized using a *GE Healthcare* ÄKTA Oligopilot plus 10 on a 20 to 50 µmol scale. After automated synthesis, RNAs were first cleaved from the solid support by treating with 3 mL of a 1:1 mixture of NH_3 aqueous solution (28% wt) and CH_3NH_2 ethanol solution (33% wt) at 55°C for 80 minutes in a tube with a sealed cap. The solid was removed by filtration and washed with 50% EtOH/H_2O. The solutions were combined and evaporated to dryness under reduced pressure. Silyl protecting groups were removed by treating the residues with 2 mL of a 1:1 (v/v) mixture of triethylamine trihydrofluoride and DMSO at 65 °C for 150 minutes in a tube with a sealed cap. After brief cooling at -32 °C, 40 mL of cold 50 mM $NaClO_4$ in acetone was added to the solution to precipitate the oligoribonucleotides. The resulting mixture was centrifuged, purified using preparative HPLC, checked using analytical HPLC, and lyophilized. The purified RNA was redissolved in 2 mL of water and passed through a *Waters* Sep-Pak C18 Cartridge with 10 g sorbent. Eluates were checked using analytical HPLC, combined, and lyophilized. The resulting white powder was stored at -32°C for future use.

Certain anticodon loops and ssRNAs are not accessible due to self-dimerization and G-quadruplex formation. Refer to Fig. S15 for a detailed discussion. The anticodon loop and ssRNA may potentially form a four-base pairing complex. See Fig. S16 for details.

Isothermal titration calorimetry experiment for anticodon loop(s) and ssRNA binding

Isothermal titration calorimetry (ITC) experiments were performed on a *Malvern* MicroCal iTC 200 at 10°C. Anticodon loops and tri-/hexamer ssRNA were dissolved in binding buffer (50 mM HEPES pH 7.2, 100 mM NaCl, 100 mM $MgCl_2$). In a typical experiment, an anticodon loop solution (800 µM, 42 µL)

was injected 28 times at 200-second intervals from a syringe into the sample cell containing ssRNA (40 μM, 350 μL) at a stirring speed of 750 RPM. The first injection was 0.5 μL of anticodon loop solution, and the subsequent 27 injections were 1.5 μL each time. The control experiment was conducted by titrating the anticodon loop with the blank buffer. The data were analyzed by MicroCal PEAQ-ITC and plotted using GraphPad Prism.

Biolayer interferometry experiment for anticodon loop and ssRNA binding

Biolayer interferometry (BLI) experiments were performed on a *FortéBio* Octet RED384 system with streptavidin biosensors at 20°C. The biotin-labelled ssRNA (0.9 μM) was diluted in the same buffer as used in the ITC experiments, i.e. 50 mM HEPES pH 7.2, 100 mM NaCl, 100 mM $MgCl_2$, and bound to the streptavidin biosensor for 180 s, followed by a washing step (60 s) with ITC buffer, an association step (180 s or 300 s) of the anticodon loops (0 to ca. 800 μM) to the sensor-bound ssRNA, and a dissociation step (180 s or 300 s) in the buffer. The assay was performed in a solid black 96-/384-well plate, using an agitation set at 1000 rpm. The control experiment was conducted by dipping the biosensor with ssRNA into the blank buffer. The data were analyzed by Data Analysis HT V11.0 and plotted using GraphPad Prism.

Fig. S1 Stacked ^{1}H-NMR spectra showing the peptidyl products of the high concentration reaction of glycyl-adenosine-5'-*O*-methylphosphate (415 mM). Identities were confirmed by serial spiking of triglycine, diglycine and DKP, whilst glycine was identified by its characteristic peak shift. Yields of 6% diglycine, 12% diketopiperazine (DKP), and < 0.5% triglycine were observed.

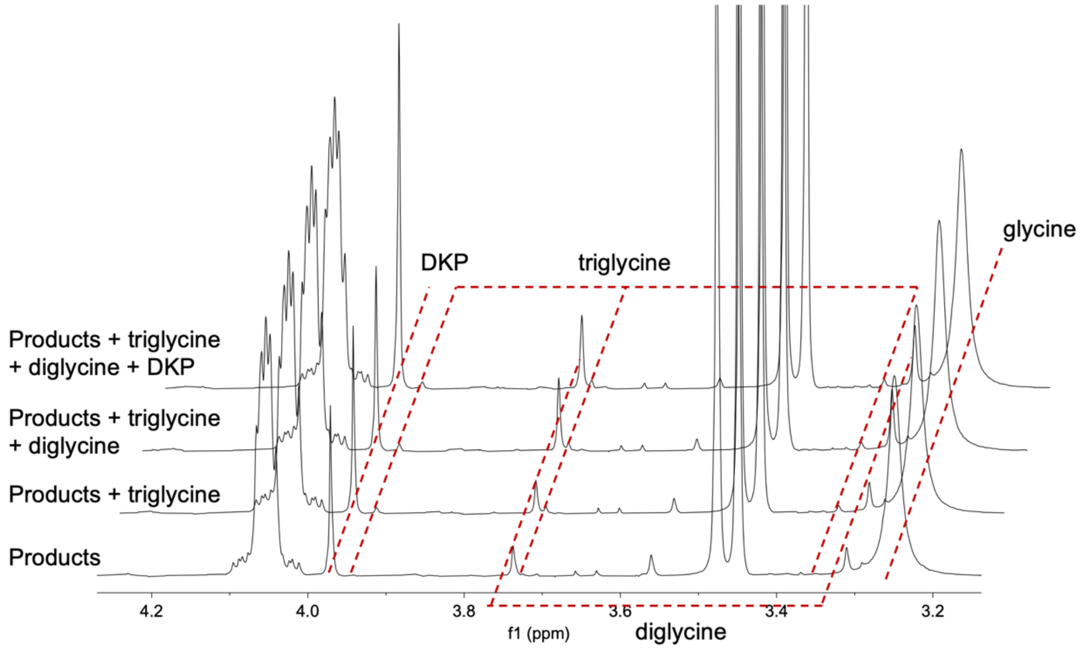

Fig. S2 Stacked ^{1}H-NMR spectra showing the peptidyl products of the high concentration reaction of free glycine and glycyl-adenosine-5'-*O*-methylphosphate (both 207 mM). Identities were confirmed by serial spiking of triglycine, diglycine and DKP, whilst glycine was identified by its characteristic peak shift. Yields of 3.4% diglycine, 3% diketopiperazine (DKP), and < 0.1% triglycine were observed.

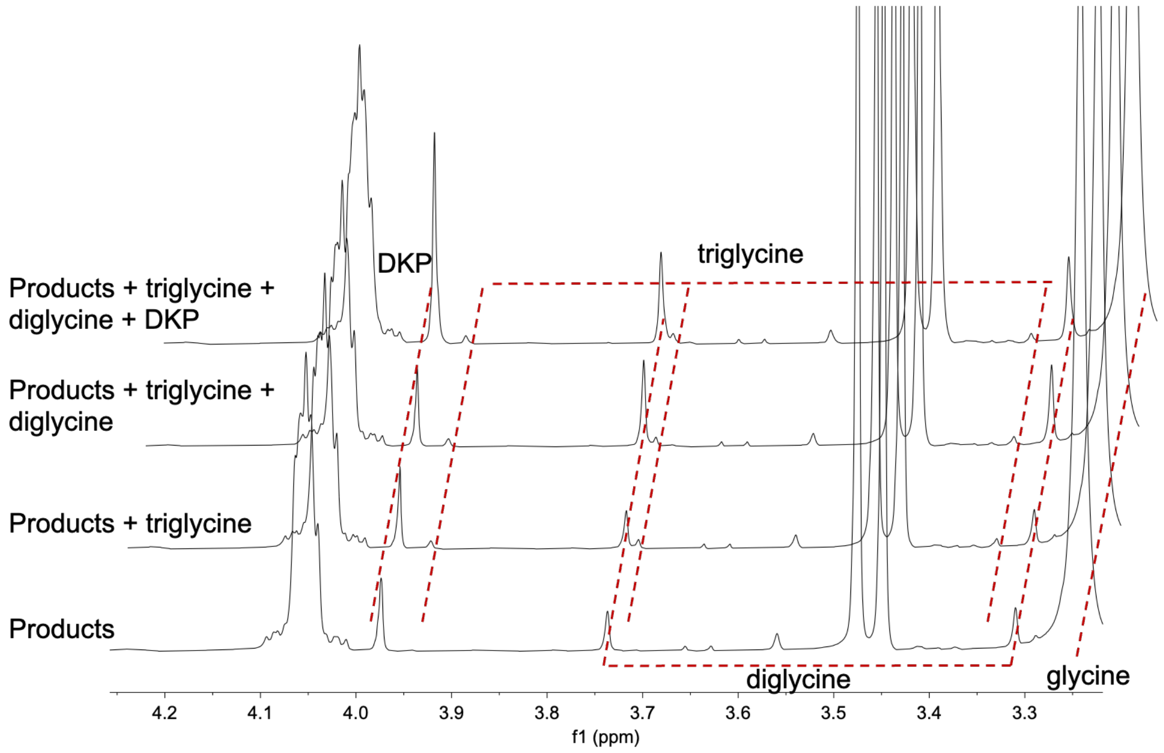

Fig. S3 HPLC chromatograms of the products of peptide bond formation after dansylation. Top: 11.3 – 39.6 min. Bottom: 14.96 – 20.72 min at increased intentisty. A) Standard mix of glycine, diglycine and triglycine; B) products of glycyl-adenosine-5'-*O*-methylphosphate at 415 mM (refer to Fig. S1); C) products of glycyl-adenosine-5'-*O*-methylphosphate and free glycine at 207 mM (refer to Fig. S2); D) products of glycyl-adenosine-5'-*O*-methylphosphate at 35 mM (refer to Fig. S4); E) products of glycyl-adenosine-5'-*O*-methylphosphate and formyl-glycyl-adenosine-5'-*O*-methylphosphate at 207 mM (refer to Fig. S7). Detection wavelength: 300 nm.

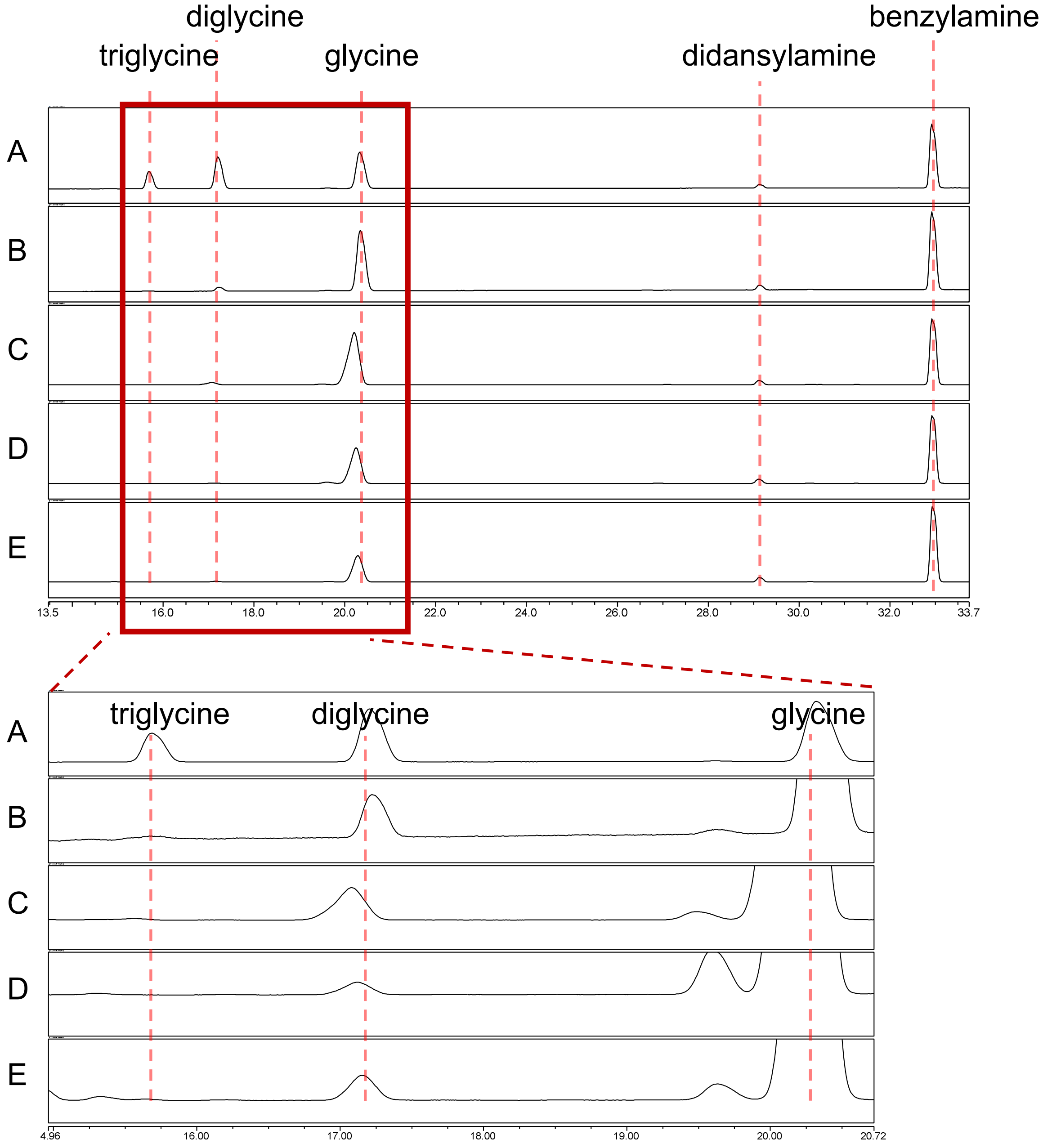

Fig. S4 Stacked ^{1}H-NMR spectra showing the peptidyl products of the low concentration reaction of glycyl-adenosine-5'-*O*-methylphosphate (35 mM). Identities were confirmed by serial spiking of diglycine and DKP, whilst glycine was identified by its characteristic peak shift. Traces of DKP, but no diglycine were observed.

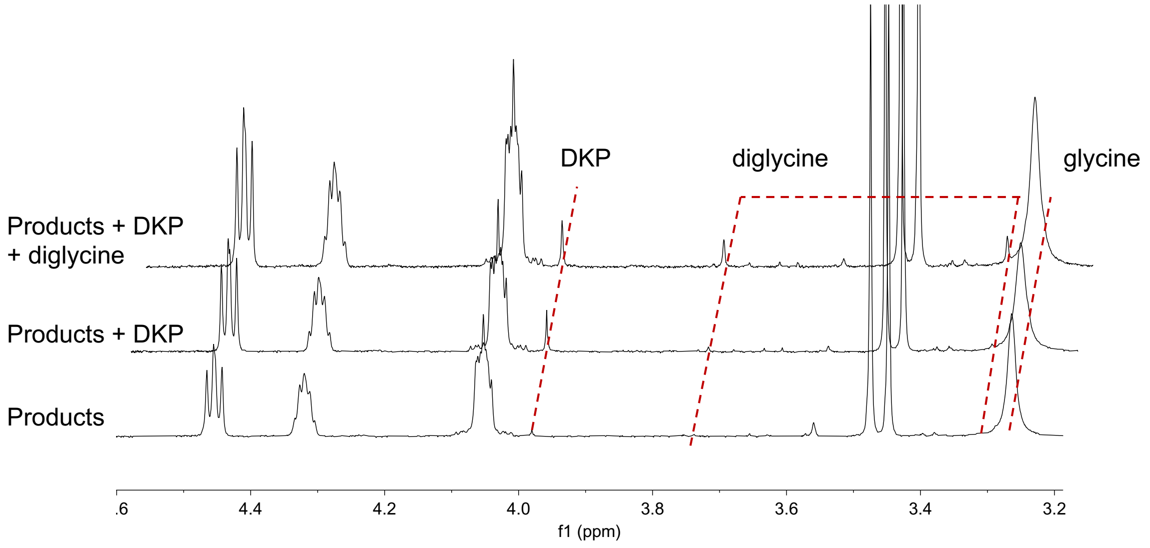

Fig. S5 Yields and kinetic date of the hydrolysis of diglycyl-adenosine-5'-*O*-methylphosphate. Conditions: adenylate (10 mM), pH = 8.2, 25 °C.

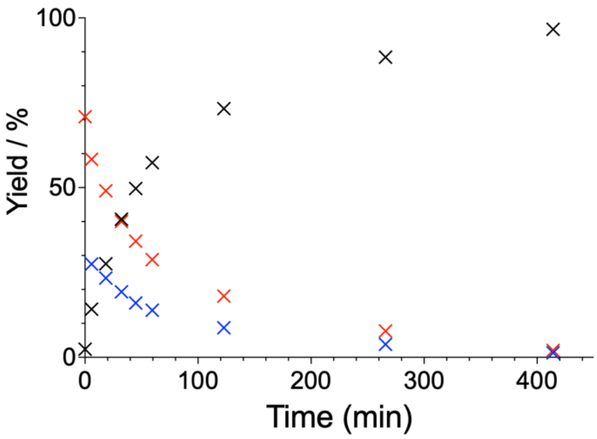

× 6.08 ppm, Adenosine-5'-*O*-methylphosphate

× 6.18 ppm, 3'-diglycyl-adenosine-5'-*O*-methylphosphate

× 6.35 ppm, 2'-diglycyl-adenosine-5'-*O*-methylphosphate

Fig. S6 Stacked ^{1}H-NMR spectra between 5.15 – 6.60 ppm (top) and 3.25 – 4.55 ppm (bottom) for the half life of hydrolysis of diglycyl-adenosine-5'-*O*-methylphosphate. After 16 hours the ratio of DKP and diglycine was calculated from the normalised integration of 3.98 ppm (DKP) and 3.75 ppm (diglycine) to be 3:1.

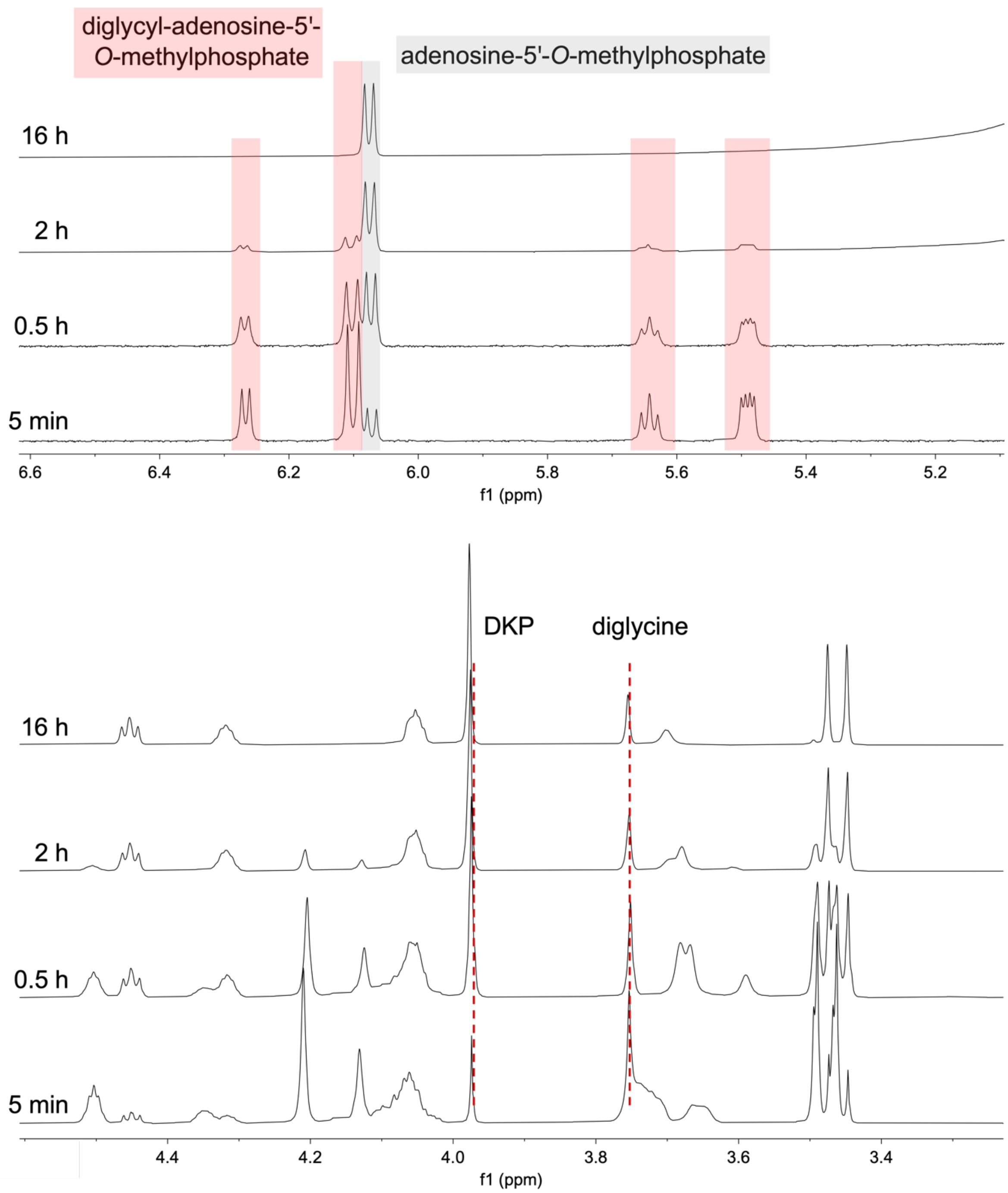

Fig. S7 Stacked ^{1}H-NMR spectra showing the peptidyl products of the high concentration reaction of formyl-glycyl-adenosine-5'-*O*-methylphosphate (207 mM) and free glycyl-adenosine-5'-*O*-methylphosphate (207 mM). Identities were confirmed by serial spiking of triglycine, diglycine and DKP, whilst glycine was identified by its characteristic peak shift. Yield of 1.5% diglycine, 5.4% *N*-formyl-digly, 5.2% DKP, ~0.1% tripeptides are observed. F-, formyl group.

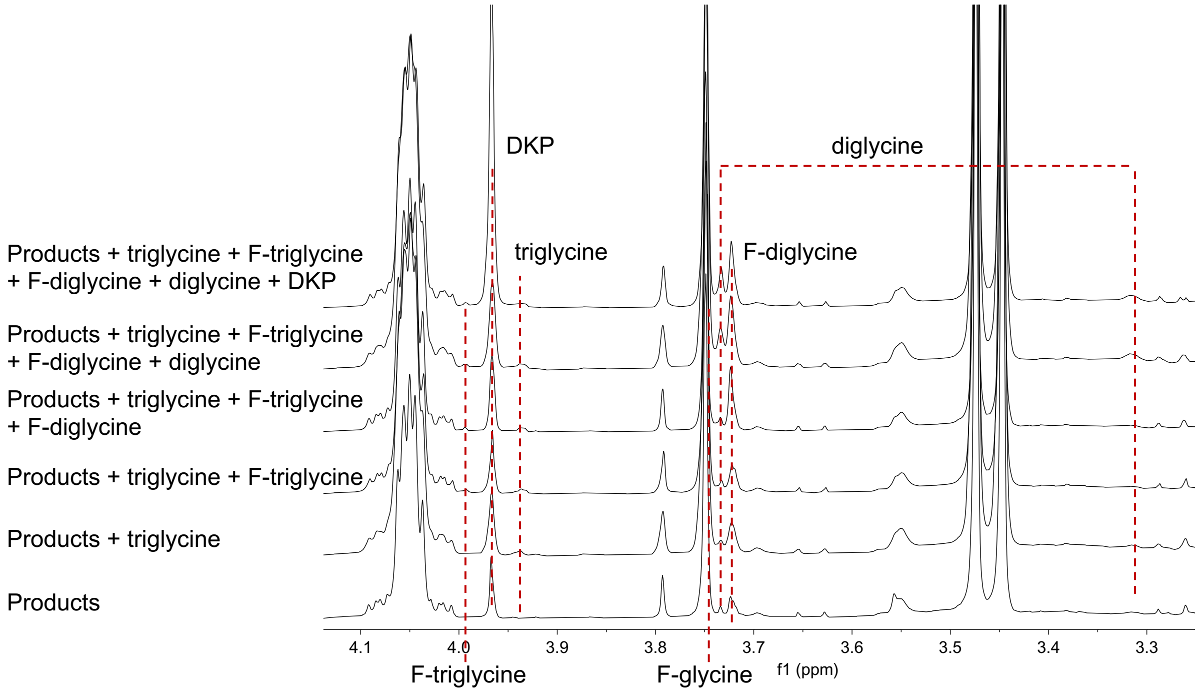

Fig. S8 Isothermal titration calorimetry curves of anticodon loops with varied residue at positions 33, 35, and 37 (consensus tRNA numbering). It is documented that the 2'-hydroxyl group of U33 forms a hydrogen bond with N7 of A35 (A). In the case of G35, a bifurcated hydrogen bond bridges the (U33)O2'-H and guanine N7 and O6. The hydrogen bond is less obvious when residue 35 is a pyrimidine (C or U). Titration of the loop 5'-CUGGUAA to codon 5'-ACC revealed a K_D of 5.7 μM (B). Replacing the second G of the anticodon to C abrogated the binding (C). A parallel example showed that loop 5'-CUCGCAA bound to 5'-GCG with a K_D of 1.1 μM. Altering the U33 to A decreased the affinity to 6.0 μM (D,E). These results confirmed that U33:R35 hydrogen bond enhances the binding of the anticodon loop to ssRNA. In addition, A37 contributes towards the tight binding through base stacking as shown by the reduction in binding when it is changed to C (F,G). Conditions: 100 mM NaCl, 100 mM $MgCl_2$, 50 mM HEPES, pH 7.2, 10 ºC.

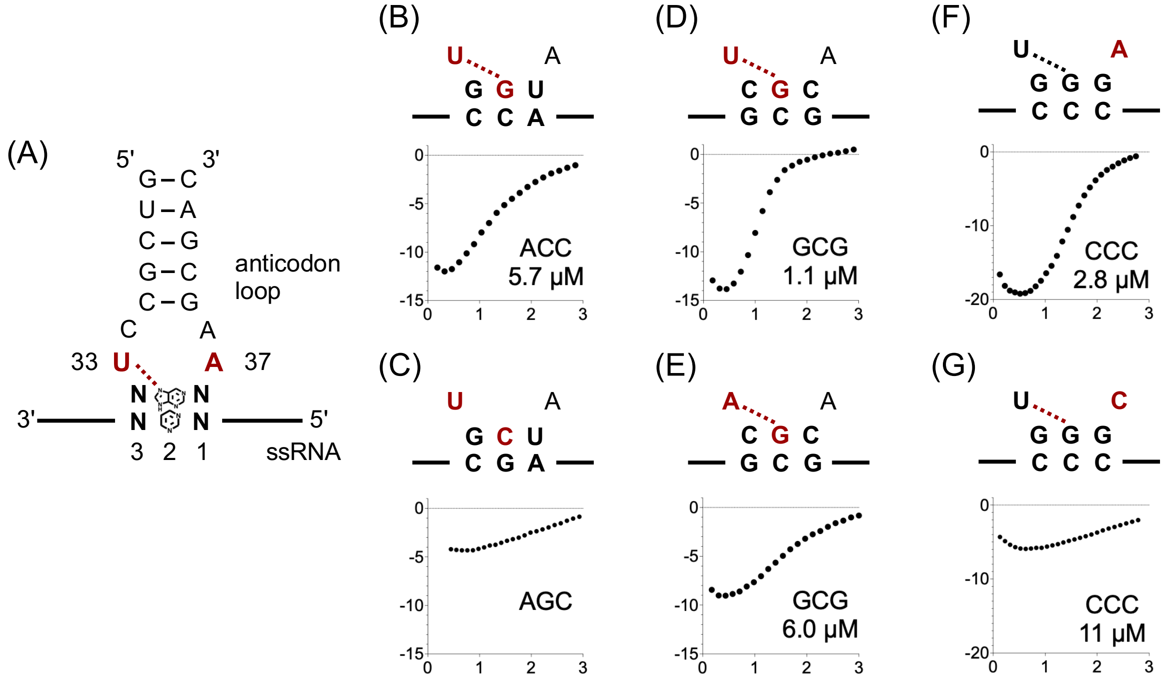

Fig. S9 Measurement of anticodon loop:codon binding affinity using BLI. (A) Schematic representation of an anticodon loop binds to a biotin-labelled ssRNA containing the respective codon; (B) typical BLI binding curves of four codons, two family-box codons in black (top, CACCC and AGUCA), and two split-box codons in red (below, CCAUU and AUUCC). Conditions: 0–800 μM each anticodon loop, 100 mM NaCl, 100 mM $MgCl_2$, 50 mM HEPES, pH 7.2, 20 °C.

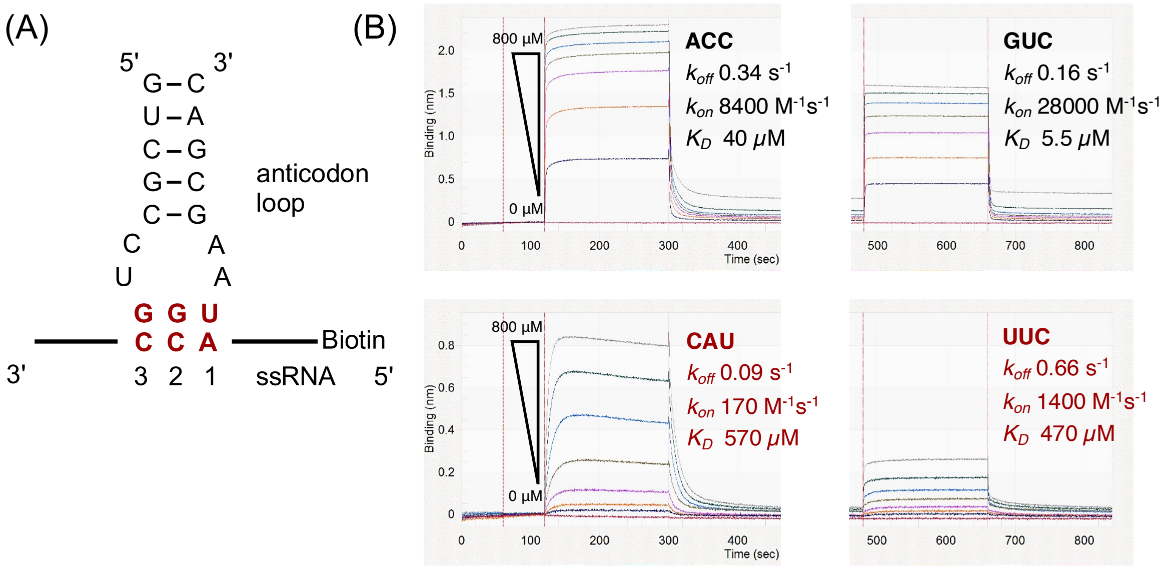

Fig. S10 Box and Whisker plots of (A) equilibrium dissociation constants, (B) association constants, and (C) disocation constants of measured anticodon loop:codon ending with A/C/G/U in two codon classes (family and split boxes). The plot offset on the right shows comparasion for all family and split box codons. P-value of permutation test for Pearson correlation coefficient is displayed. For family-box codons: NNA, n = 6; NNC, n = 8; NNG, n = 6; NNU, n =8. For split-box codons: NNA, n = 3; NNC, n = 7; NNG, n = 8; NNU, n = 8. Sequences with an n value less than 8 result from certain sequences exhibiting a binding affinity weaker than detectable by BLI (K_D > 3000).

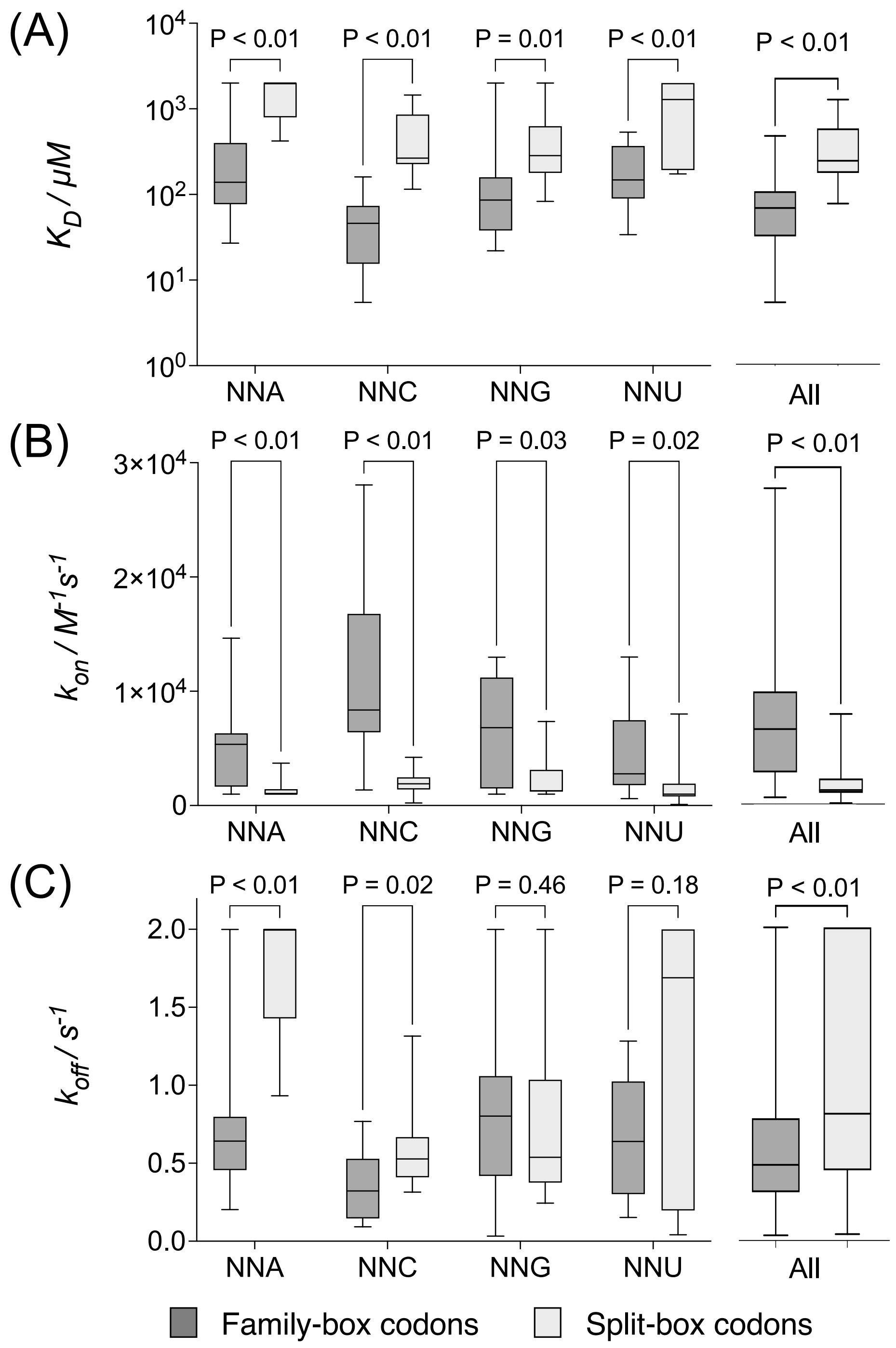

Fig. S11 Isothermal titration calorimetry curves of anticodon loops with A/C/G/U-ending codons binding to a ssRNA containing either a single triplet (light grey or red) or two continguous identical triplets (black or red). A) Codons ending in A; B) Codons ending C; C) Codons ending in G; D) Codons ending in U. Black curves represent family-box codons; red curves represent split-box codons. Empty circles in GCC represent an exothermic process due to ssRNA self-dimer and are excluded from the calculation. Titration of hexamer guanine is absent due to the self-aggregation of oligo guanine in the excluded buffer. Conditions: 800 μM anticodon loop, 40 μM ssRNA, 100 mM NaCl, 100 mM $MgCl_2$, 50 mM HEPES, pH 7.2, 10 ºC. Numerical values for K_D, molar ratio (R), and enthalpy change (kcal/mol) can be found in tables S8 and S9.

A) Codons ending in A

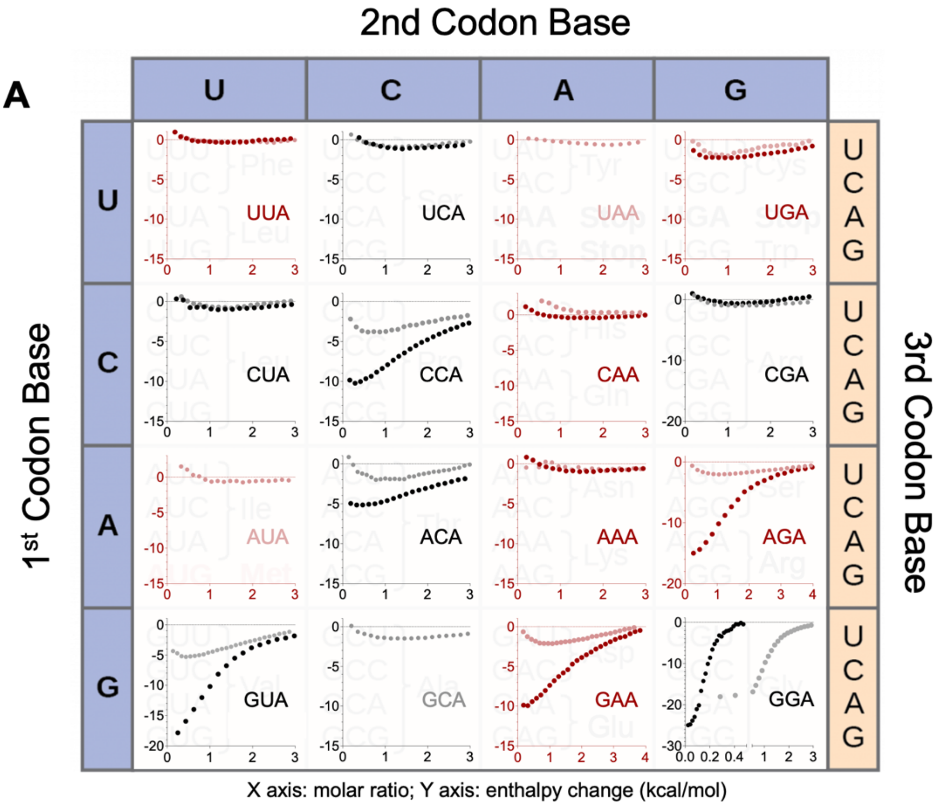

B) Codons ending in C

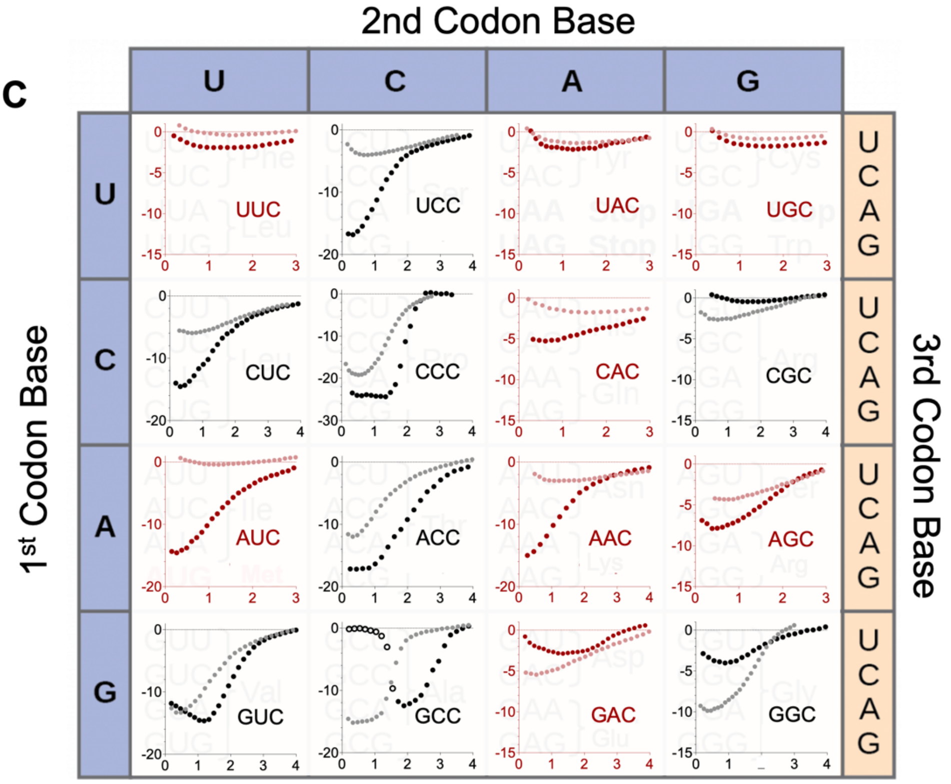

X axis: molar ratio; Y axis: enthalpy change (kcal/mol)

C) Codons ending in G

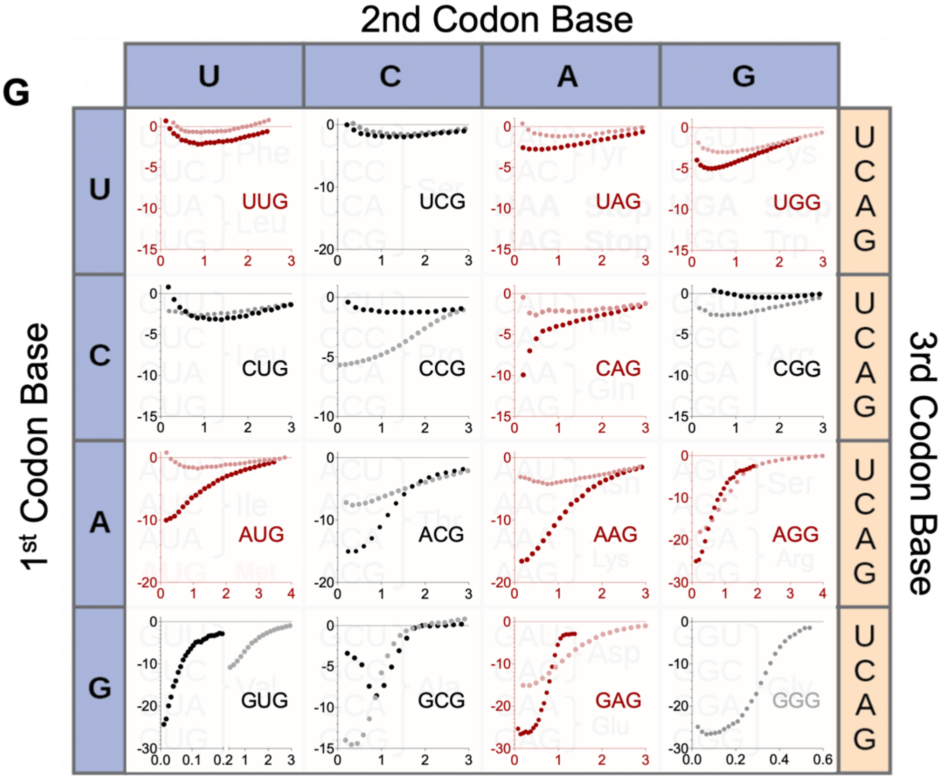

X axis: molar ratio; Y axis: enthalpy change (kcal/mol)

D) Codons ending in U

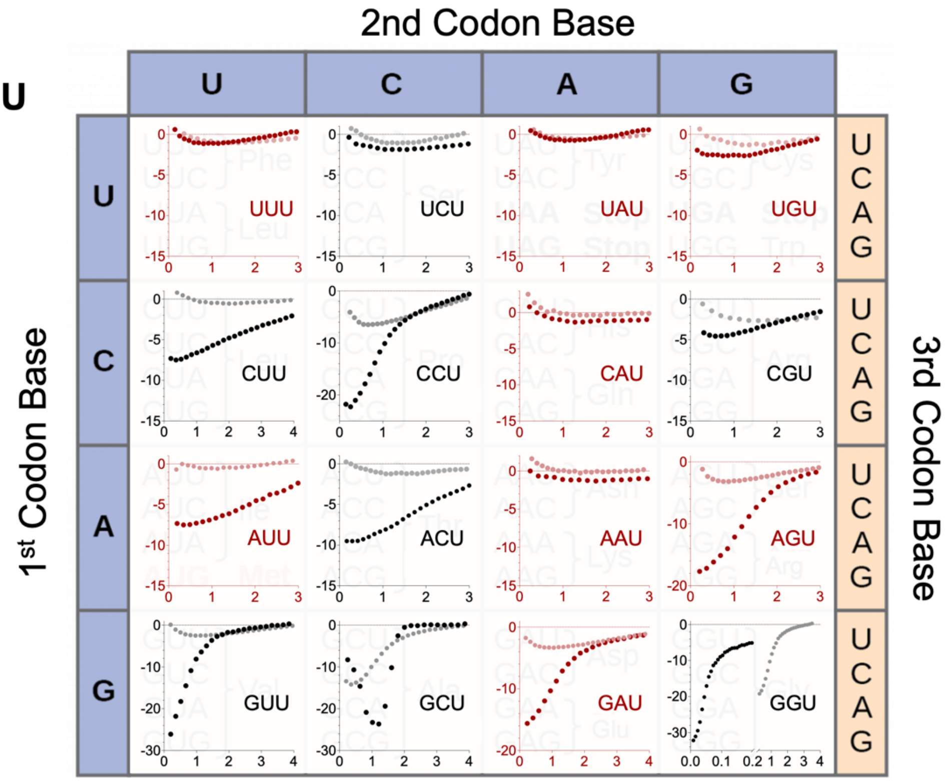

X axis: molar ratio; Y axis: enthalpy change (kcal/mol)

Fig. S12 Isothermal titration calorimetry curves of family-box anticodon loop interacting with another family-box loop. Conditions: 800 μM anticodon loops, 100 mM NaCl, 100 mM $MgCl_2$, 50 mM HEPES, pH 7.2, 10 ºC.

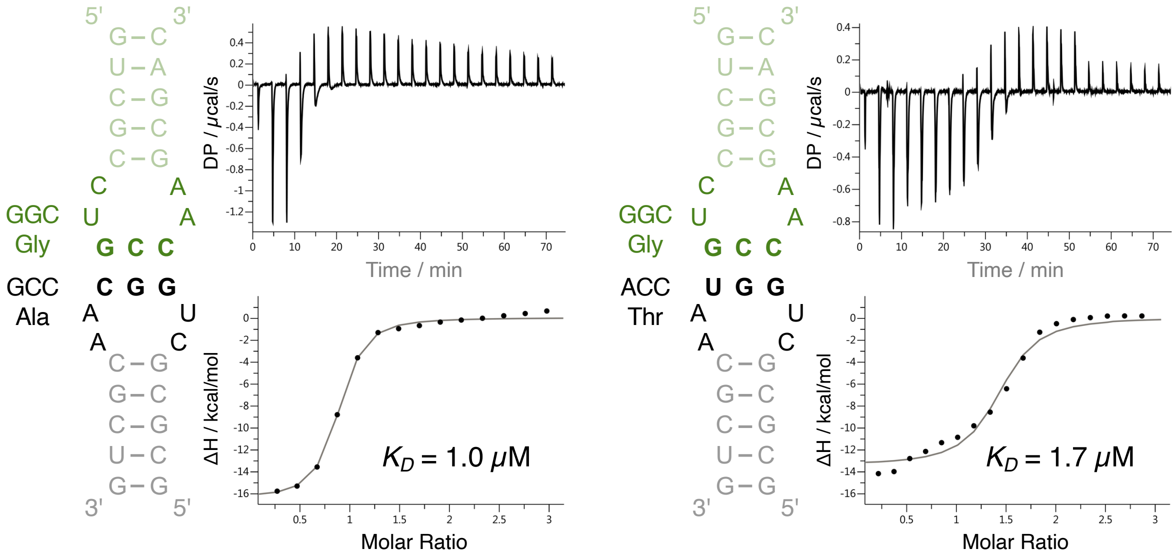

Fig. S13 BLI curves depicting the binding of ssRNAs containing either two family-box codons or one family-box (black font) and one split-box codon (blue font). Initially, the ssRNAs were saturated with one codon at a fixed concentration (200 μM). Subsequently, a second loop was added with a gradient concentration (ranging from 0 to 400 μM), whilst maintaining the first loop at the same fixed concentration. Then, the biotinylated RNA complex was moved back to the first anticodon loop solution to wash out the second loop. The binding measurement was repeated four more times for a total of five cycles. Conditions: 100 mM NaCl, 100 mM $MgCl_2$, 50 mM HEPES, pH 7.2, 20 ºC.

5'-A CCC UCC G (Pro-Ser)

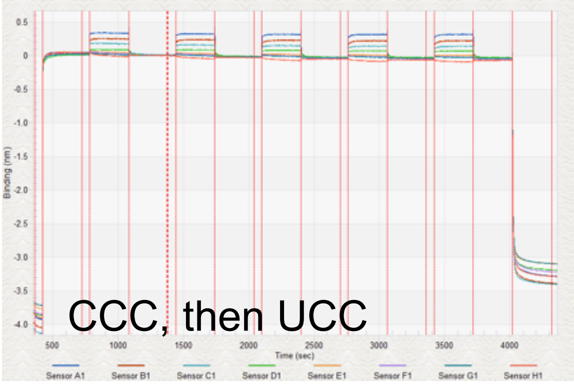

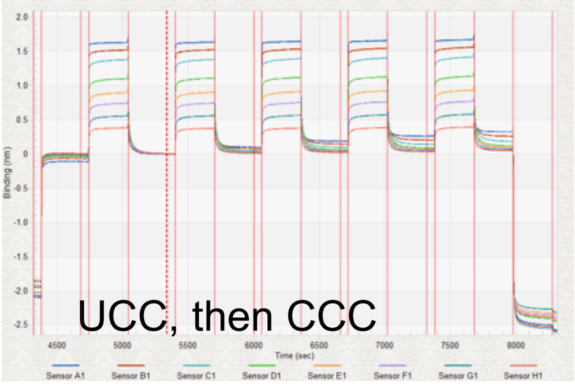

5'-A ACG GUC C (Thr-Val)

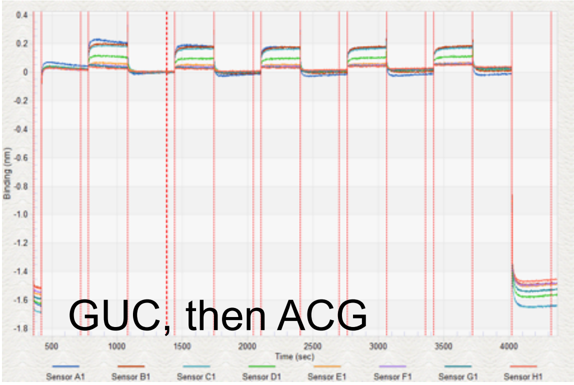

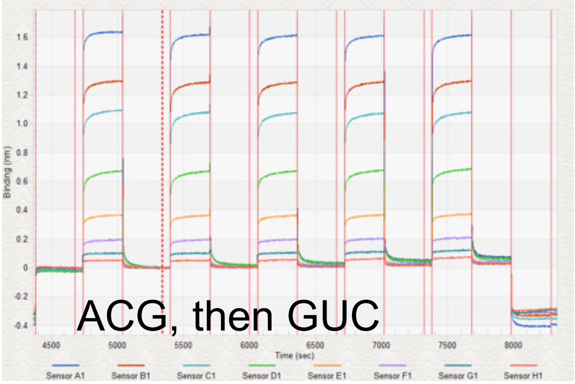

5'-A CGA GGU C (Arg-Gly)

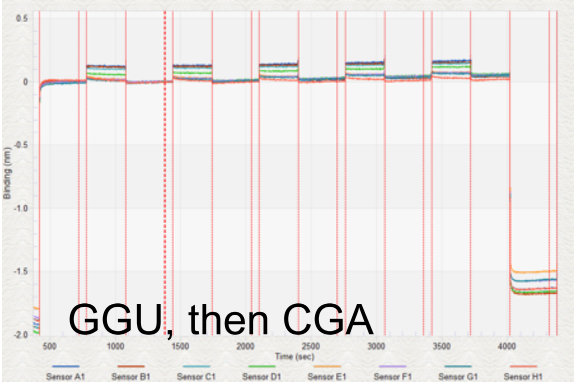

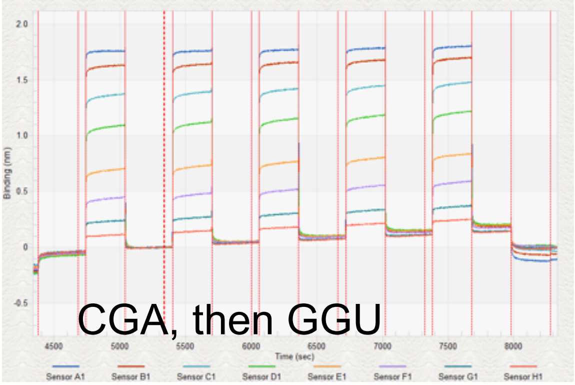

5'-A GAC GCC C (Asp-Ala)

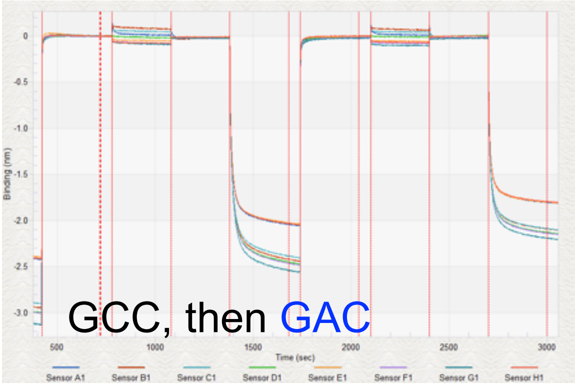

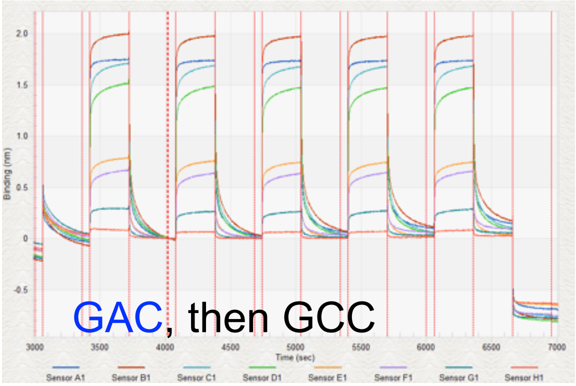

5‘-A GUC UAC C (Val-Tyr)

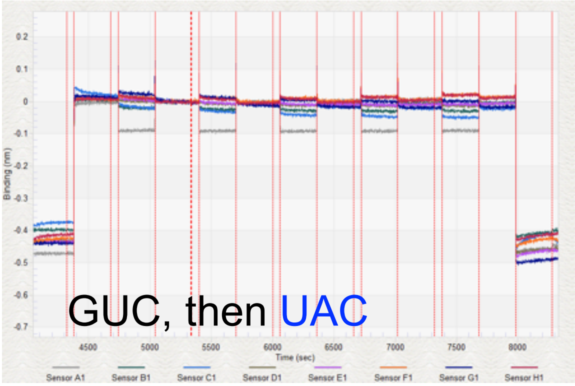

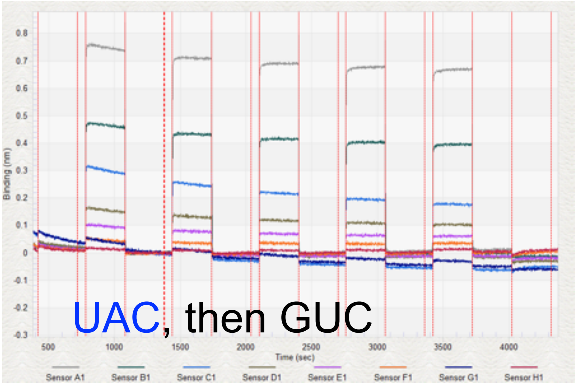

Fig. S14 Biolayer interferometery binding curves using ssRNA with two different split-box codons. (A) single anticodon loop 5'-CUGAAAA binding to UUC; (B) single anticodon loop 5'-CUAUGAA binding to CAU; (C) equally mixed two anticodon loops aim at binding to UUC and CAU. Neither hairpins binding to UUC or CAU could be included at saturating conditions due to their low binding affinity. Simultaneous binding afforded an apparent K_D of 190 μM. As this is very similar to that we recorded for CAU on its own (180 μM) we conclude only the CAU is binding. Conditions: 0–800 μM each anticodon loop, 100 mM NaCl, 100 mM $MgCl_2$, 50 mM HEPES, pH 7.2, 20 ºC.

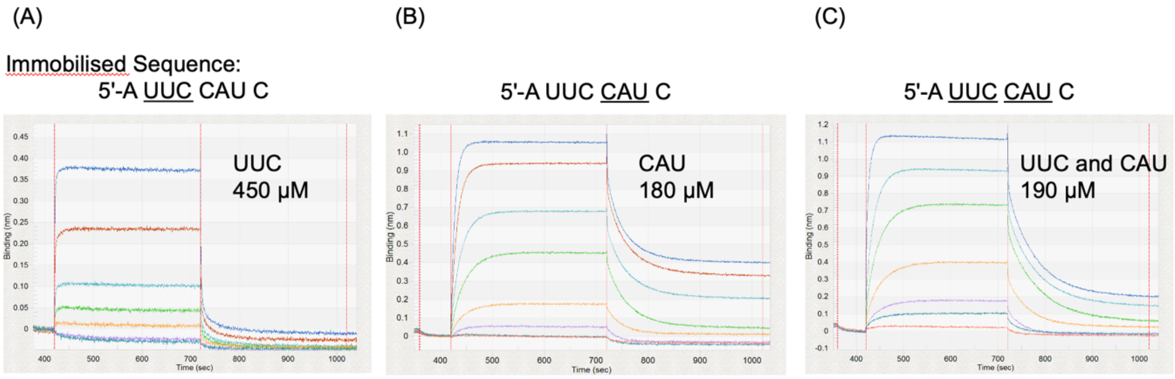

Fig. S15 Anticodon loops that are able to form self-dimers. General position of loop sequences are underlined. Bold letters indicate anticodon triplets. Anticodon loops for codons ANU, UNA, GNC, and CNG are likely to dimerize, reducing the authentic concentration for the loop in both BLI and ITC and leading to a false stronger equilibrium dissociation constants. The issue was alleviated by denaturing and quickly reannealing the anticodon loops.

```
5' GUCGC  CUANUAA  GCGAC
   |||||   || ||   |||||
3' CAGCG  AAUNAUC  CGCUG

5' GUCGC  CUUNAAA  GCGAC
   |||||   || ||   |||||
3' CAGCG  AAANUUC  CGCUG

5' GUCGC  CUGNCAA  GCGAC
   |||||   || ||   |||||
3' CAGCG  AACNGUC  CGCUG

5' GUCGC  CUCNGAA  GCGAC
   |||||   || ||   |||||
3' CAGCG  AAGNCUC  CGCUG
```

Fig. S16 Schematic representation of tRNA anticodon loop and homo-triplet binding where four bases pairing is theoretically possible. The numbering on the anticodon loop is consistent with the existing tRNA anticodon loop domain, aligning with the standard structure of a typical tRNA, which comprises 76 nucleotides.

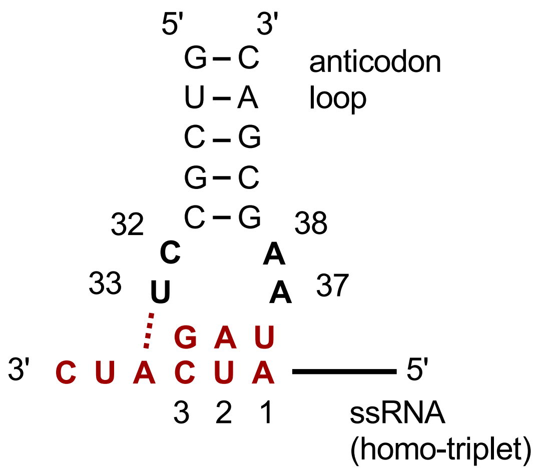

Fig. S17 Binding affinity of single anticodon-loop (5'- CUNGGAA) and trimer RNA (5'-CCN) with one mismatched base pair.

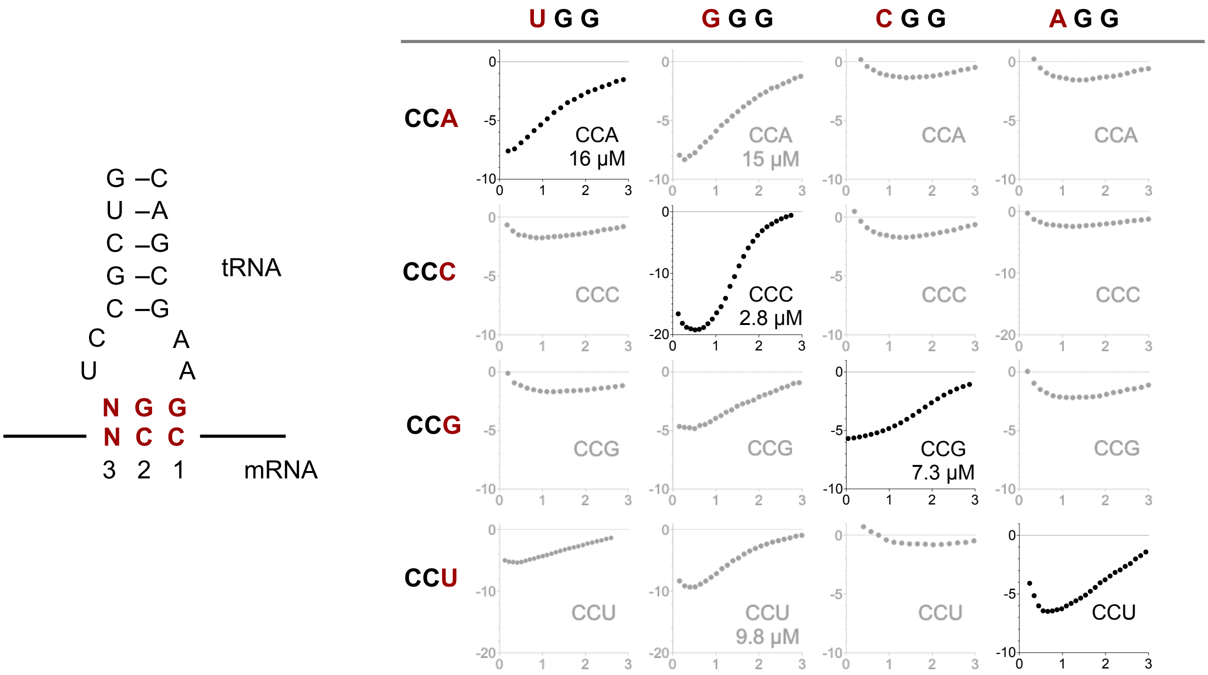

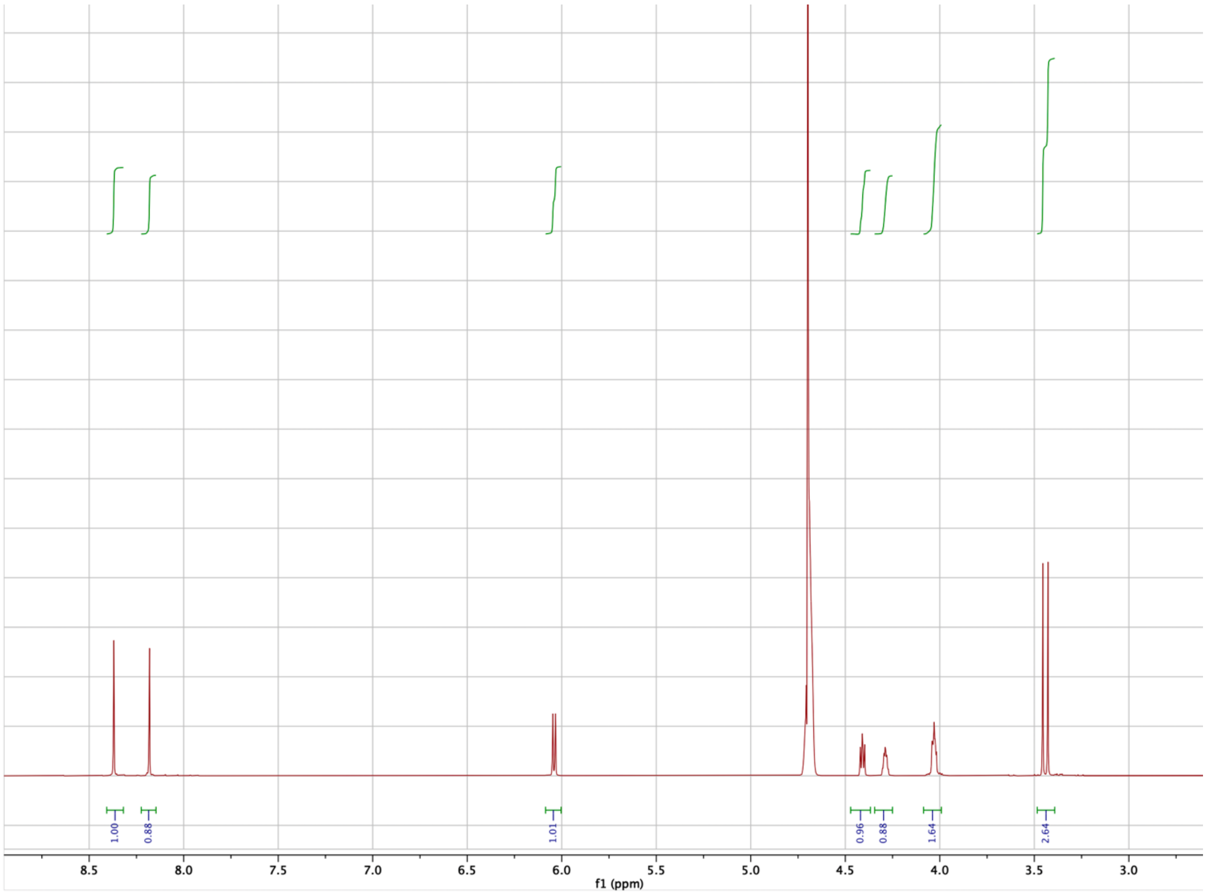

Fig. S18 ^{1}H-NMR spectrum of adenosine-5'-*O*-methylphosphate

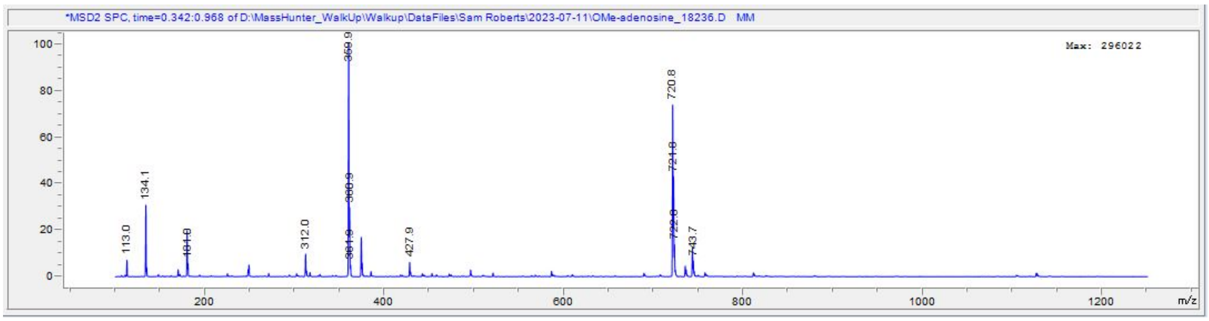

Fig. S19 Negative ion low resolution mass spectrum of adenosine-5'-*O*-methylphosphate

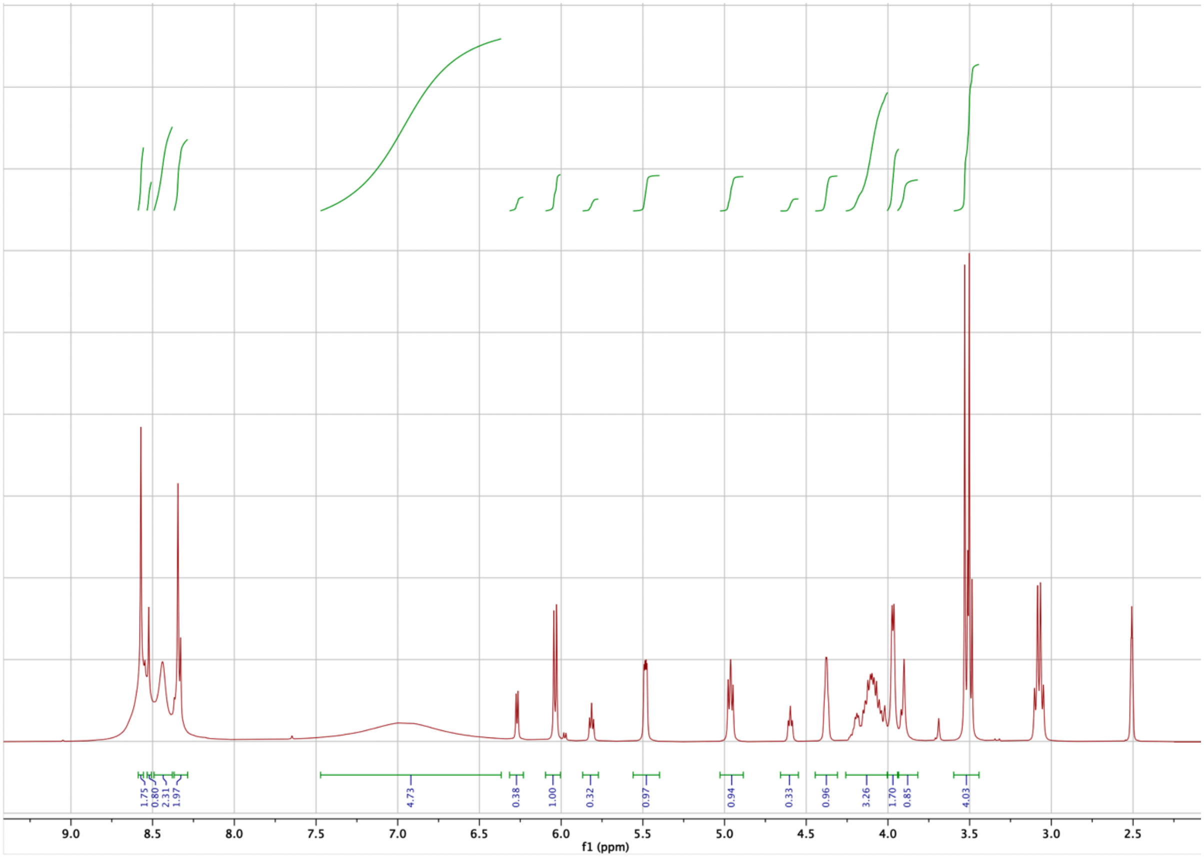

Fig. S20 ^{1}H-NMR spectrum of 2'/3'-glycyl-adenosine-5'-*O*-methylphosphate

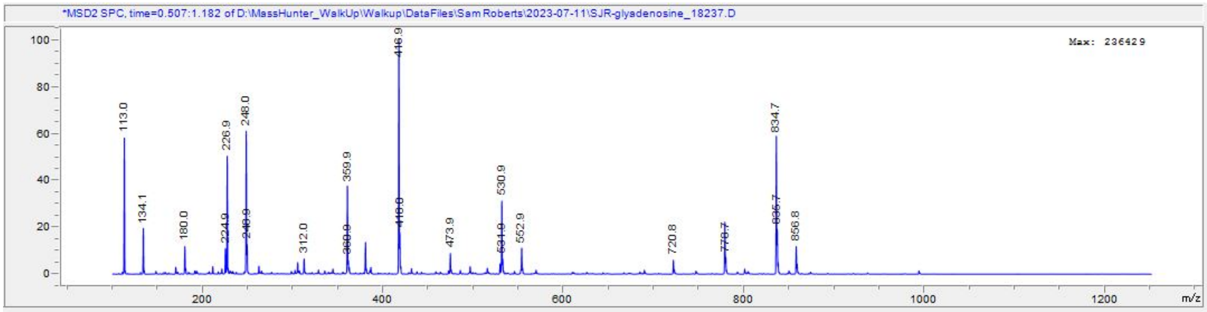

Fig. S21 – Negative ion low resolution mass spectrum of 2'/3'-glycyl-adenosine-5'-*O*-methylphosphate

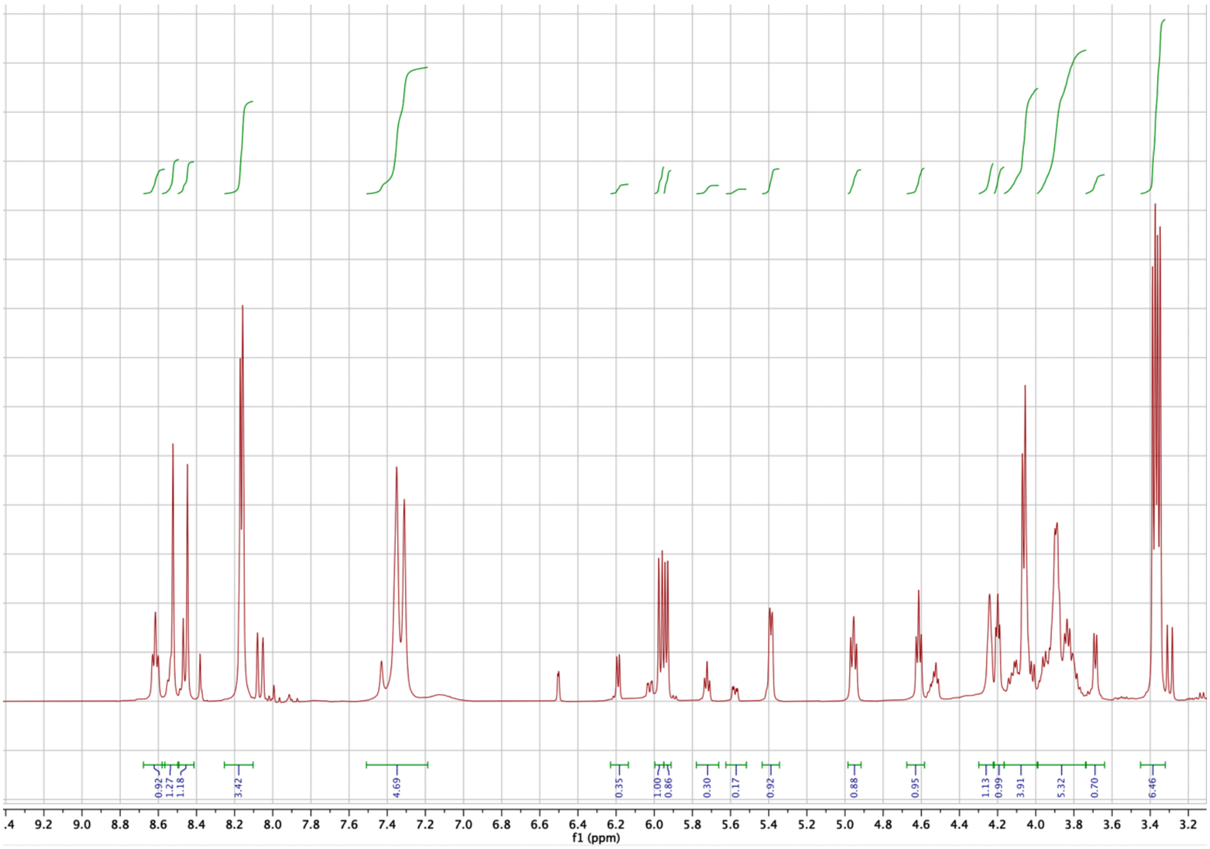

Fig. S22 ^{1}H-NMR spectrum of 2'/3'-*N*-formyl-glycyl-adenosine-5'-*O*-methylphosphate

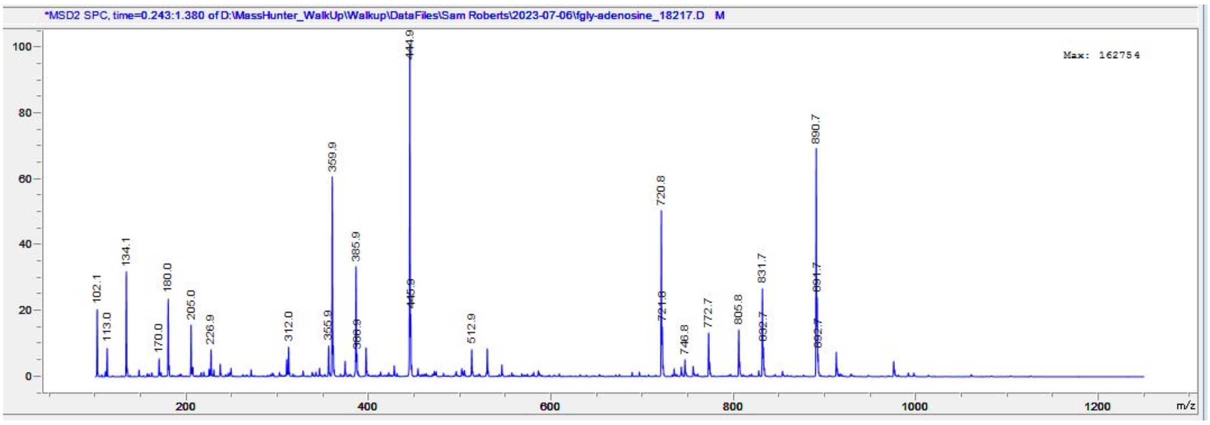

Fig. S23 Negative ion low resolution mass spectrum of 2'/3'-*N*-formyl-*g*lycyl-adenosine-5'-*O*-methylphosphate

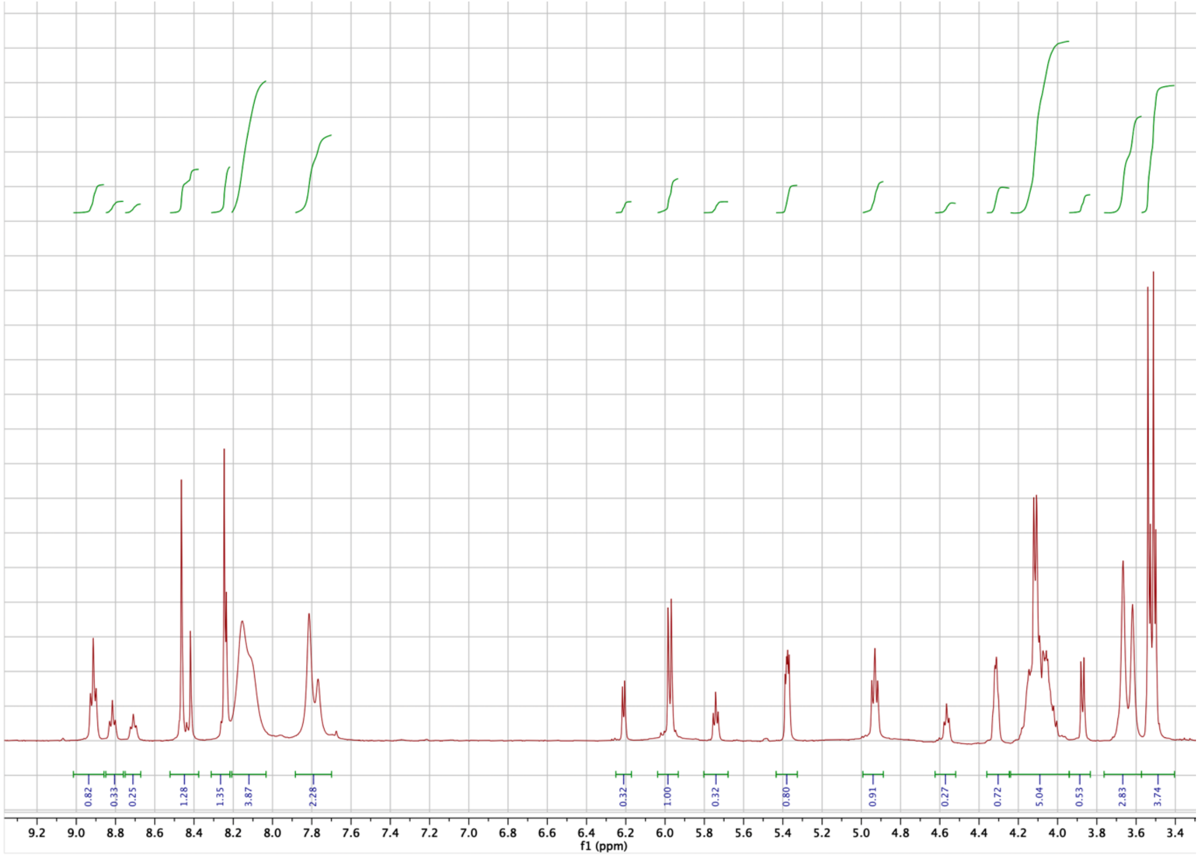

Fig. S24 ^{1}H-NMR spectrum of 2'/3'-diglycyl-adenosine-5'-*O*-methylphosphate

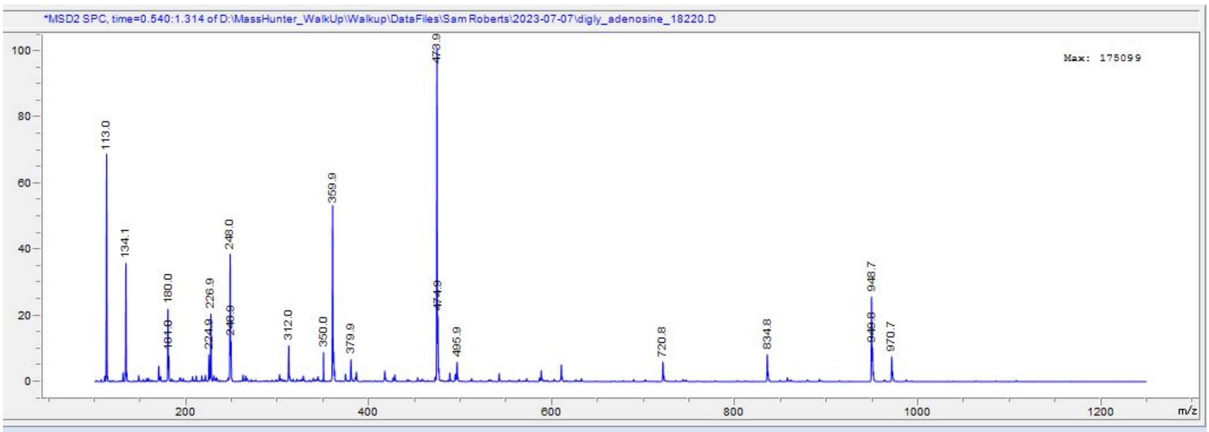

Fig. S25 Negative ion low resolution mass spectrum 2'/3'-diglycyl-adenosine-5'-*O*-methylphosphate

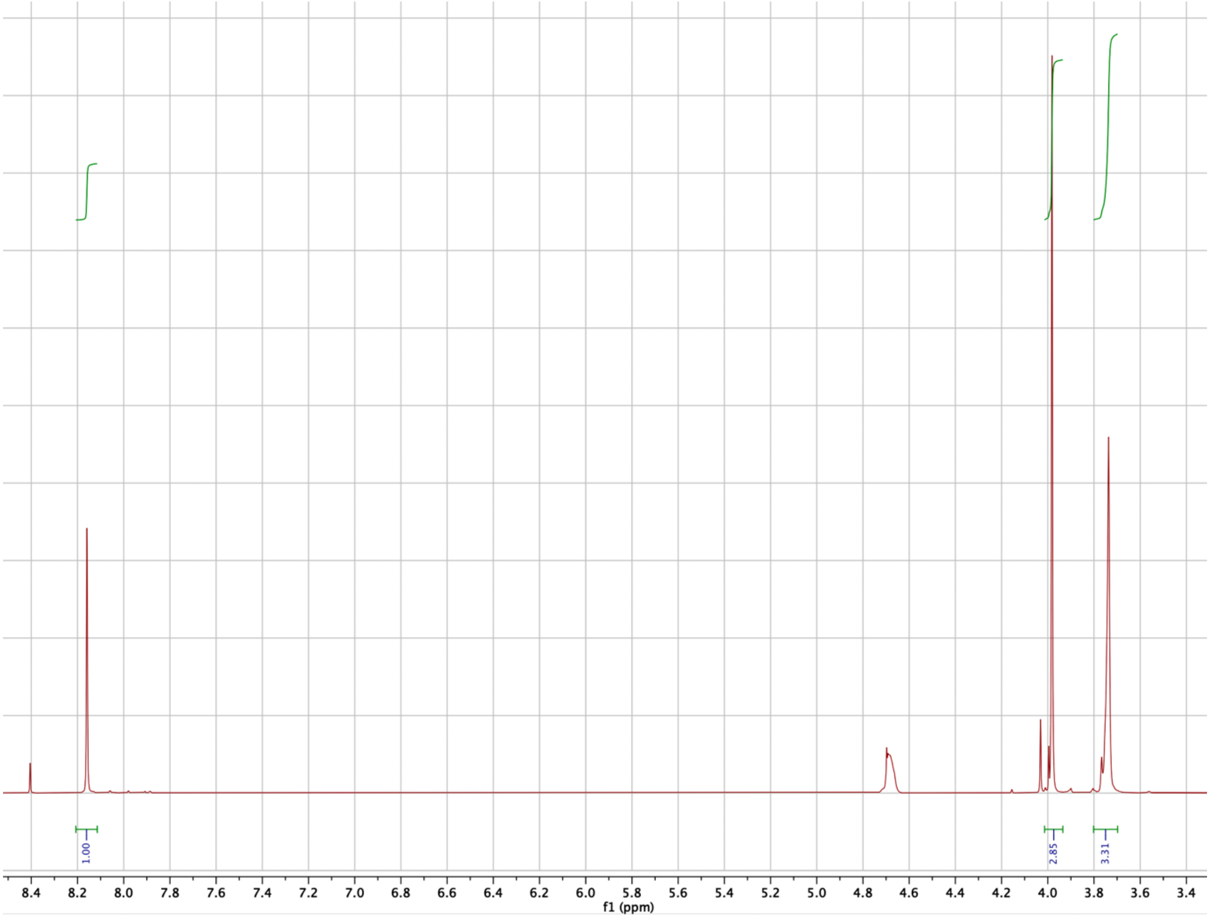

Fig. S26 ^{1}H-NMR spectrum of *N*-formyl-diglycine

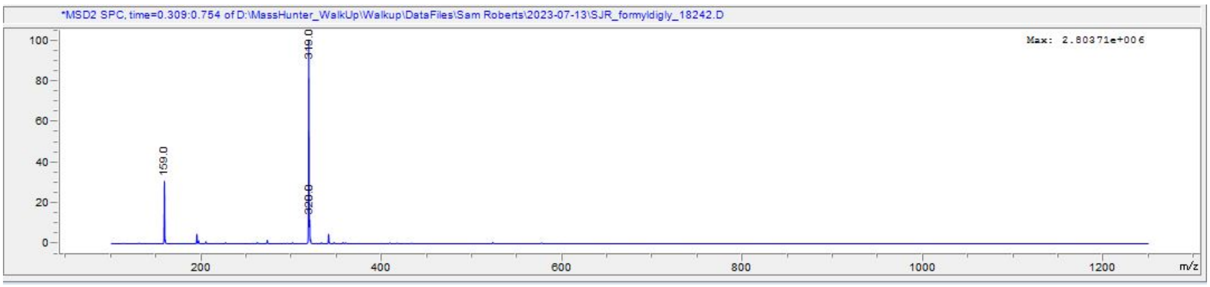

Fig. S27 Negative ion low-resolution mass spectrum *N*-formyl-diglycine

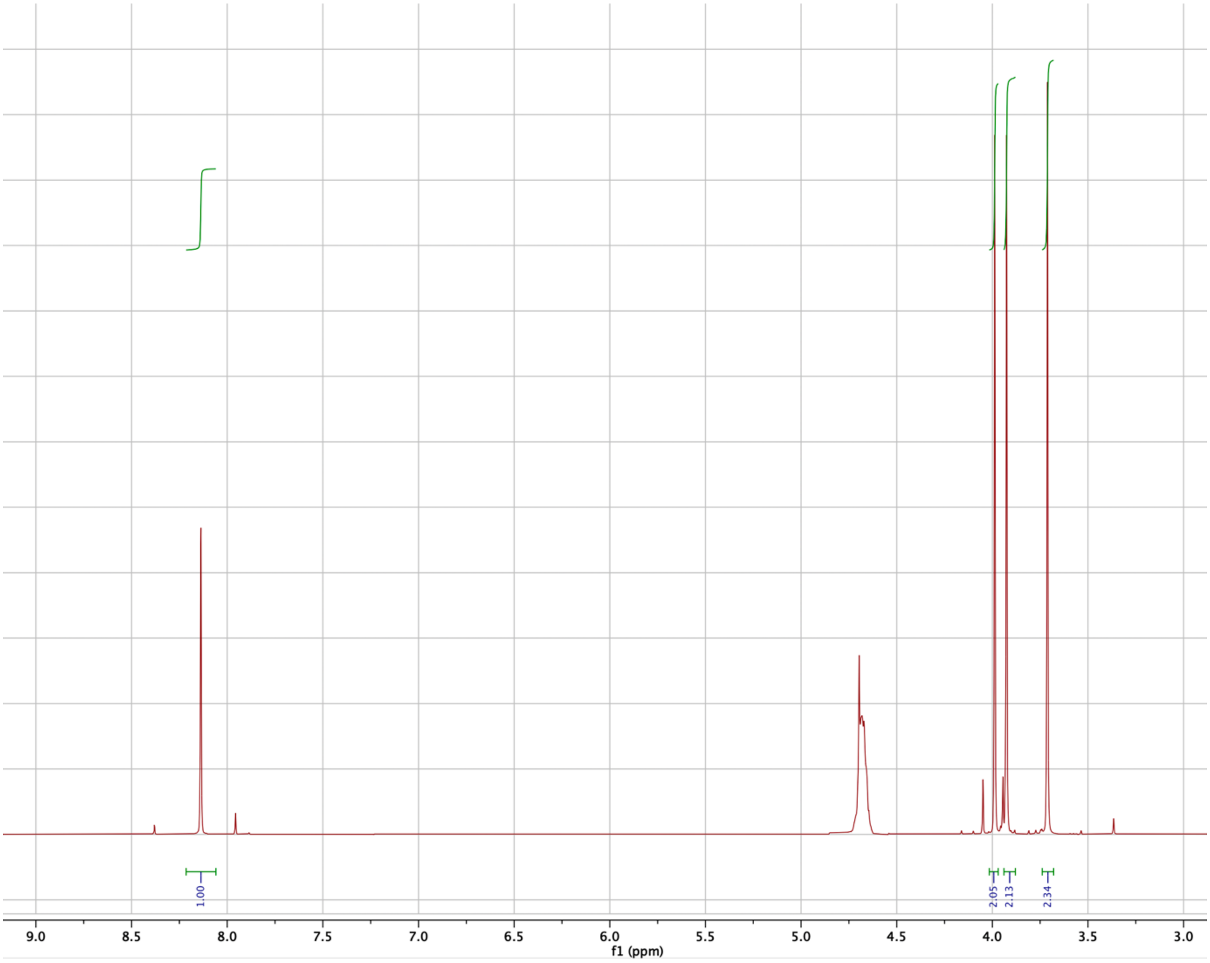

Fig. S28 ^{1}H-NMR spectrum of *N*-formyl-triglycine

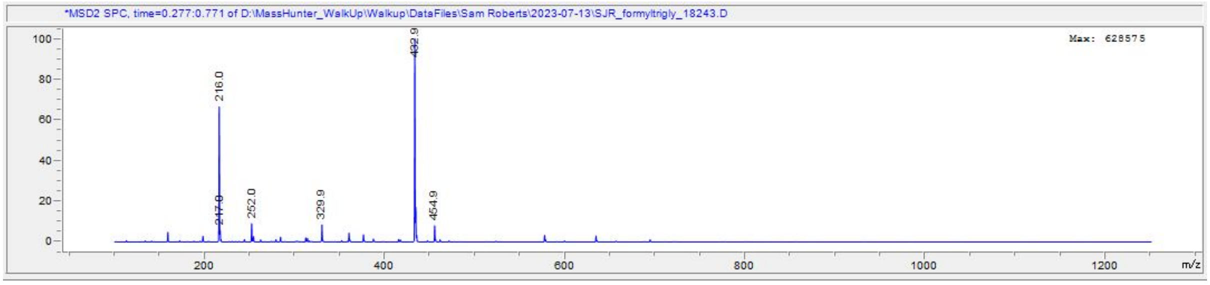

Fig. S29 – Negative ion low resolution mass spectrum *N*-formyl-triglycine

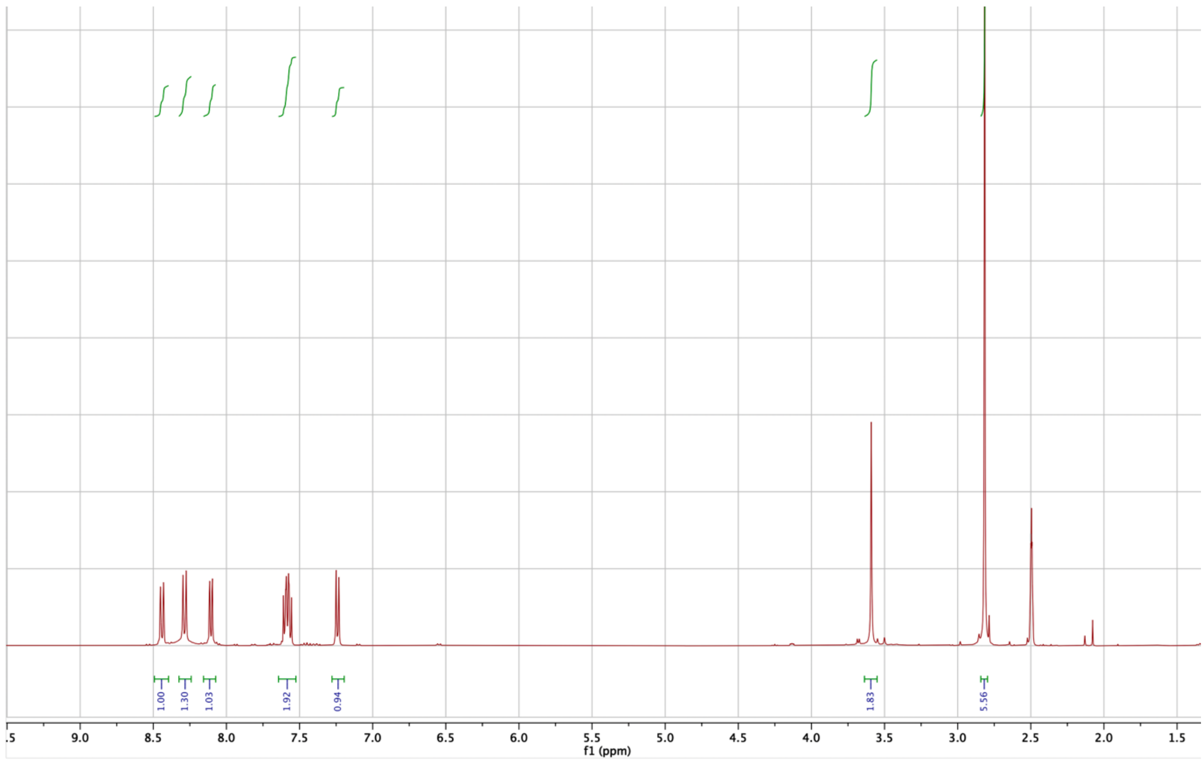

Fig. S30 ^{1}H-NMR spectrum of *N*-dansyl-glycine

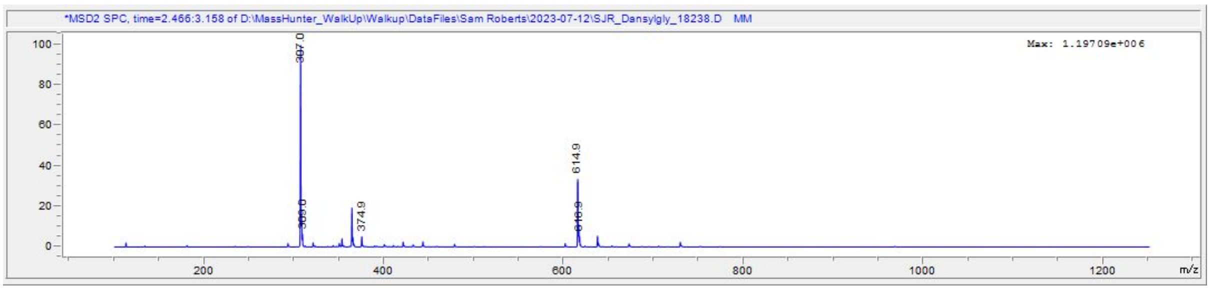

Fig. S31 Negative ion low resolution mass spectrum *N*-dansyl-glycine

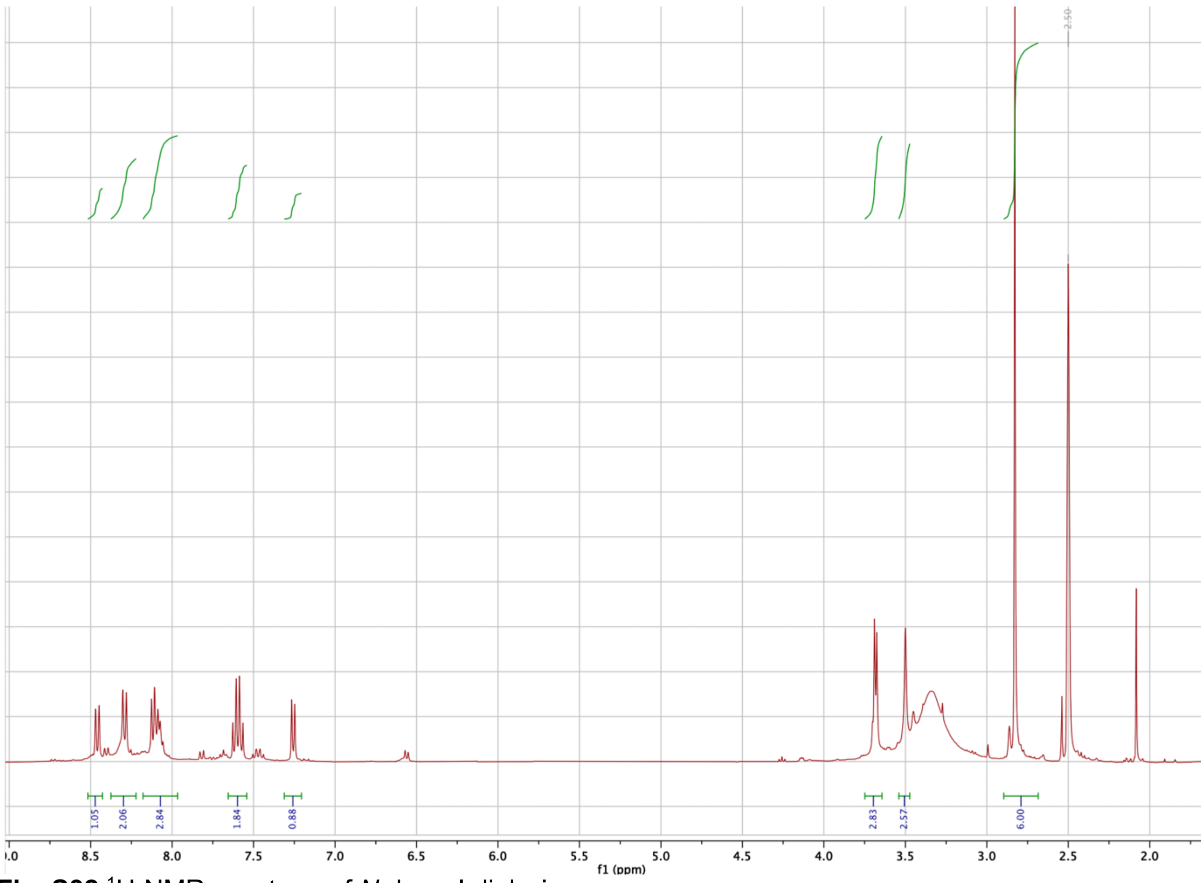

Fig. S32 ^{1}H-NMR spectrum of *N*-dansyl-diglycine

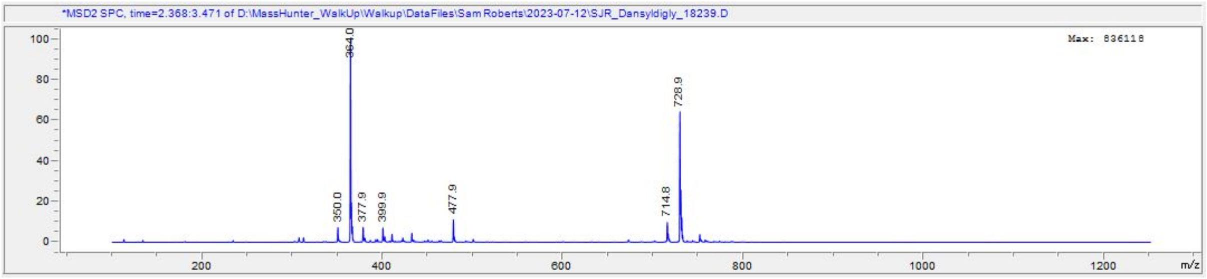

Fig. S33 Negative ion low-resolution mass spectrum *N*-dansyl-diglycine

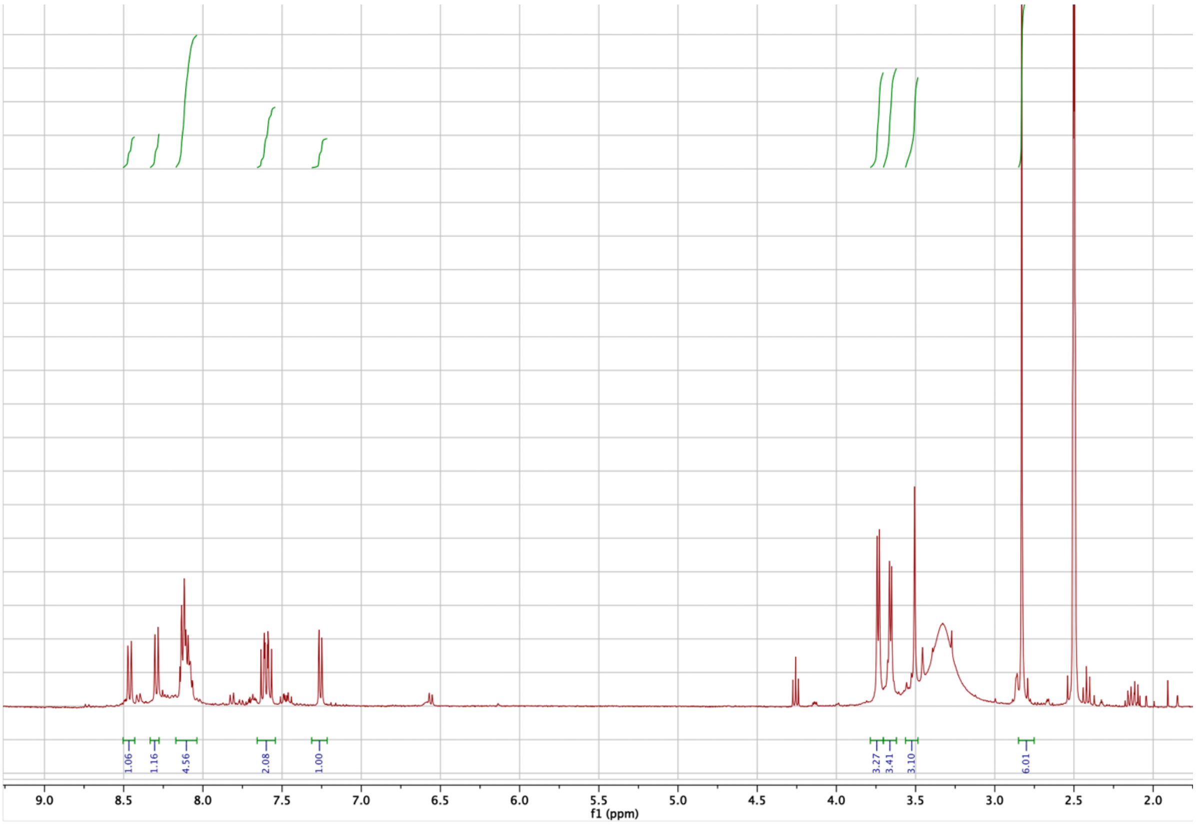

Fig. S34 ^{1}H-NMR spectrum of *N*-dansyl-triglycine

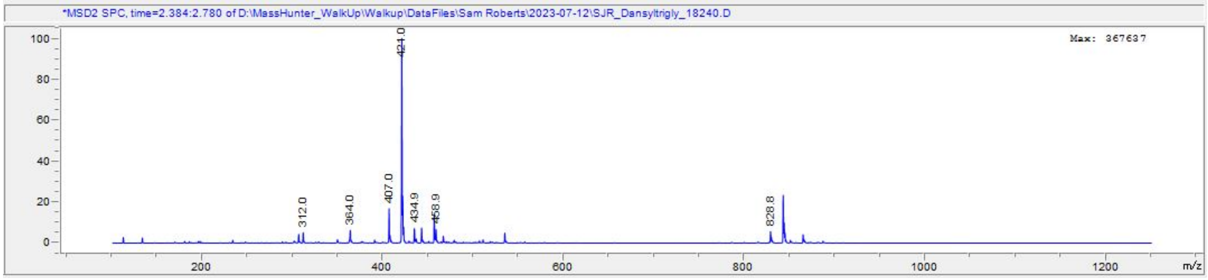

Fig. S35 Negative ion low-resolution mass spectrum *N*-dansyl-triglycine

Table S1 Comparison of the yields (%) of dansylation of a mixture of glycine, diglycine and triglycine with the observed ratio of the mixture by ^{1}H NMR.

Yields (%) By Dansylation	23.97	50.91	101.97
Expected Yields (%) By NMR	20.50	43.56	100.00

Table S2 Yields (%) relative to the total amount of glycine species from the reactions of adenylates presented in this paper. Quantified by ^{1}H NMR direct integration (blue), ^{1}H NMR integration after the addition of known amounts of standard (std., black) or by HPLC analysis post-dansyl functionalisation (red). * Lack of a nucleophilic centre (free amino group) prevents dansyl functionalisation. ** Peak could not be observed by ^{1}H NMR due to apparent resonance broadening.

Reagents	Method	Tri-Gly	Di-Gly	Gly	DKP*	Formyl -trigly*	Formyl-digly*	Formyl-gly*
Gly-adenosine (415 mM)	Dansyl	0.12	3.06	71.0	-	-	-	-
	NMR	0.36	5.64	75.7	11.8	-	-	-
	NMR std.	0.50	6.09	-	12.8	-	-	-
Gly-adenosine + Glycine (207 mM both)	Dansyl	0.05	2	63.2	-	-	-	-
	NMR	0.09	3.39	83.8	2.97	-	-	-
	NMR std.	0.09	3.32	-	3	-	-	-
Gly-adenosine (35 mM)	NMR	0.00	0.42	94.7	1.13	-	-	-
	NMR std.	0.00	0.42	-	1.26	-	-	-
Gly-adenosine + Formyl-gly-adenosine (207 mM both)	Dansyl	0.04	1.36	44.8	-	-	-	-
	NMR	0.12	1.74	-**	5.18	0.15	5.44	31.01
	NMR std.	0.64	1.80	-	5.61	0.13	5.33	-
Digly-adenosine (10mM)	NMR	-	22.7	-	77.3	-	-	-

Table S3 Synthesized anticodon loops RNAs for the BLI and ITC experiments. Found, observed (raw) mass number from LCMS in negative ion mode; Expt., the experimental mass number calculated from the observed values, i.e. Expt. = (Found + 1) × charges, 4 or 5 charges; Calc., mass number in theory. Underlined residues highlight the loop sequence.

No.	Sequence (5' -3')	Calc.	Expt.	Found
1	GUCGC CU UUU AA GCGAC	5360.7	5360.0	1071.3
2	GUCGC CU GUU AA GCGAC	5399.8	5400.4	1349.1
3	GUCGC CU CUU AA GCGAC	5359.8	5360.0	1071.1
4	GUCGC CU AUU AA GCGAC	5383.8	5384.5	1075.9
5	GUCGC CU UGU AA GCGAC	5399.8	5401.0	1079.2
6	GUCGC CU GGU AA GCGAC	5438.8	5439.2	1358.8
7	GUCGC CU CGU AA GCGAC	5398.8	5400.0	1079.0
8	GUCGC CU AGU AA GCGAC	5422.8	5424.0	1083.8
9	GUCGC CU UCU AA GCGAC	5359.8	5360.0	1071.0
10	GUCGC CU GCU AA GCGAC	5398.8	5398.5	1078.7
11	GUCGC CU CCU AA GCGAC	5358.8	5360.0	1071.0
12	GUCGC CU ACU AA GCGAC	5382.8	5383.0	1075.6
13	GUCGC CU UAU AA GCGAC	5383.8	5384.5	1075.9
14	GUCGC CU GAU AA GCGAC	5422.8	5423.6	1354.9
15	GUCGC CU CAU AA GCGAC	5382.8	5383.5	1075.7
16	GUCGC CU AAU AA GCGAC	5406.8	5407.5	1080.5
17	GUCGC CU UUG AA GCGAC	5399.8	5400.0	1079.0
18	GUCGC CU GUG AA GCGAC	5438.8	5439.6	1358.9
19	GUCGC CU CUG AA GCGAC	5398.8	5399.5	1078.9
20	GUCGC CU AUG AA GCGAC	5422.8	5423.5	1083.7
21	GUCGC CU UGG AA GCGAC	5438.8	5439.5	1086.9
22	GUCGC CU GGG AA GCGAC	5477.8	5478.5	1094.7
23	GUCGC CU CGG AA GCGAC	5437.8	5438.8	1358.7
24	GUCGG CU AGG AA CCGAC	5461.8	5463.0	1091.6
25	GUCGC CU UCG AA GCGAC	5398.8	5399.5	1078.9
26	GUCGC CU GCG AA GCGAC	5437.8	5439.5	1086.9
27	GUCGC CU CCG AA GCGAC	5397.8	5399.0	1078.8
28	GUCGC CU ACG AA GCGAC	5421.8	5421.5	1083.3
29	GUCGC CU UAG AA GCGAC	5422.8	5423.0	1083.6
30	GUCGC CU GAG AA GCGAC	5461.8	5462.5	1091.5
31	GUCGC CU CAG AA GCGAC	5421.8	5421.5	1083.3
32	GUCGC CU AAG AA GCGAC	5445.8	5446.5	1088.3
33	GUCGC CU UUC AA GCGAC	5359.8	5360.5	1071.1
34	GUCGC CU GUC AA GCGAC	5398.8	5399.6	1348.9
35	GUCGC CU CUC AA GCGAC	5358.8	5359.6	1338.9
36	GUCGC CU AUC AA GCGAC	5382.8	5383.0	1075.6
37	GUCGG CU UGC AA CCGAC	5398.8	5399.5	1078.9

38	GUCGC CU GGC AA GCGAC	5437.8	5438.4	1358.6
39	GUCGC CU CGC AA GCGAC	5397.8	5398.0	1348.5
40	GUCGC CU AGC AA GCGAC	5421.8	5422.5	1083.5
41	GUCGC CU UCC AA GCGAC	5358.8	5358.5	1070.7
42	GUCGC CU GCC AA GCGAC	5397.8	5399.0	1078.8
43	GUCGC CU CCC AA GCGAC	5357.8	5359.0	1070.8
44	GUCGC CU ACC AA GCGAC	5381.8	5382.0	1075.4
45	GUCGC CU UAC AA GCGAC	5382.8	5383.2	1344.8
46	GUCGC CU GAC AA GCGAC	5421.8	5422.0	1083.4
47	GUCGC CU CAC AA GCGAC	5381.8	5382.4	1344.6
48	GUCGC CU AAC AA GCGAC	5405.8	5407.0	1080.4
49	GUCGC CU UUA AA GCGAC	5383.8	5384.5	1075.9
50	GUCGC CU GUA AA GCGAC	5422.8	5422.5	1083.5
51	GUCGC CU CUA AA GCGAC	5382.8	5383.5	1075.7
52	GUCGC CU AUA AA GCGAC	5406.8	5407.5	1080.5
53	GUCGC CU UGA AA GCGAC	5422.8	5424.0	1083.8
54	GUCGC CU GGA AA GCGAC	5461.8	5462.0	1364.5
55	GUCGC CU CGA AA GCGAC	5421.8	5423.0	1083.6
56	GUCGC CU AGA AA GCGAC	5445.8	5446.5	1088.3
57	GUCGC CU UCA AA GCGAC	5382.8	5383.5	1075.7
58	GUCGC CU GCA AA GCGAC	5421.8	5422.8	1354.7
59	GUCGC CU CCA AA GCGAC	5381.8	5381.0	1075.2
60	GUCGC CU ACA AA GCGAC	5405.8	5407.0	1080.4
61	GUCGC CU UAA AA GCGAC	5406.8	5407.6	1350.9
62	GUCGC CU GAA AA GCGAC	5445.8	5446.8	1360.7
63	GUCGC CU CAA AA GCGAC	5405.8	5406.5	1080.3
64	GUCGC CU AAA AA GCGAC	5429.8	5429.5	1084.9

Table S4 Purchased biotin-labelled oligoribonucleotides for BLI experiments. In most cases, the first and final nucleosides of the octamer were fixed to adenosine and cytosine and were not used as a part of the codon.

No.	Sequence (5' -3')
400	Biotin-C UUC CAU U
401	Biotin-A GUC ACC C
402	Biotin-A UUC CAU C
403	Biotin-A GCC CUC C
404	Biotin-A GUU UCU C
405	Biotin-A GUA UCG C
406	Biotin-A GUG CUU C
407	Biotin-A GCA ACU C
408	Biotin-A GCG ACA C
409	Biotin-A GAU GGU C
410	Biotin-A GAG UUA C
411	Biotin-A GGC CGU C
412	Biotin-A GGA CGC C
413	Biotin-A GGG CUA C
414	Biotin-A CUG UUG C
415	Biotin-A AUA AGC C
416	Biotin-A AAU UAC C
417	Biotin-A AGA CAG C
418	Biotin-A UAG AAA C
419	Biotin-A UGA AGG C
420	Biotin-A CGG UCA C
421	Biotin-A GCA CGG C
422	Biotin-A CGG GCU C
423	Biotin-A GGA UCG C
424	Biotin-A UGA UAG C
425	Biotin-A CCG UCC C
426	Biotin-A GAC GAA U
427	Biotin-C GAC GAA C
428	Biotin-A GGU CUU C
429	Biotin-A CCA GCG U
430	Biotin-A CGA GGU C
431	Biotin-A ACG GUC C
432	Biotin-A CCC UCC G
433	Biotin-A GAC GCC C
434	Biotin-A GUC UAC C

Table S5 Synthesized trinucleotides (mono-triplets) and purchased hexanucleotides (homo-triplets) for the ITC experiments. Found, observed (raw) mass number from LCMS in negative ion mode; Expt., the experimental mass number calculated from the observed values, i.e. Expt. = Found + 1; Calc., mass number in theory.

No.	Triplet	Calc.	Expt.	Found	Homo-triplet (5' -3')
1	AAA	925.2	924.9	923.9	AAA AAA C
2	AAC	901.2	901.0	900.0	AAC AAC C
3	AAG	941.2	941.0	940.0	AAG AAG U
4	AAU	902.2	902.0	901.0	AAU AAU U
5	ACA	901.2	901.0	900.0	ACA ACA U
6	ACC	877.2	876.9	875.9	C ACC ACC
7	ACG	917.2	917.0	916.0	ACG ACG
8	ACU	878.2	878.0	877.0	ACU ACU U
9	AGA	941.2	940.9	939.9	AGA AGA U
10	AGC	917.2	917.0	916.0	C AGC AGC
11	AGG	957.2	956.9	955.9	AGG AGG U
12	AGU	918.2	917.9	916.9	AGU AGU U
13	AUA	902.2	902.0	901.0	AUA AUA U
14	AUC	878.2	878.0	877.0	C AUC AUC
15	AUG	918.2	917.9	916.9	G AUG AUG
16	AUU	879.2	878.9	877.9	AUU AUU U
17	CAA	901.2	900.9	899.9	CAA CAA UU
18	CAC	877.2	877.0	876.0	CAC CAC C
19	CAG	917.2	916.9	915.9	CAG CAG C
20	CAU	878.2	877.9	876.9	CAU CAU C
21	CCA	877.2	877.0	876.0	CCA CCA UU
22	CCC	853.2	853.0	852.0	CCC CCC
23	CCG	893.2	892.1	891.1	G CCG CCG
24	CCU	854.2	853.9	852.9	CCU CCU UU
25	CGA	917.2	917.0	916.0	CGA CGA C
26	CGC	893.2	892.9	891.9	CGC CGC U
27	CGG	933.2	933.0	932.0	CGG CGG
28	CGU	894.2	893.9	892.9	CGU CGU UU
29	CUA	878.2	877.9	876.9	CUA CUA C
30	CUC	854.2	853.9	852.9	CUC CUC CC
31	CUG	894.2	893.9	892.9	G CUG CUG
32	CUU	855.1	854.9	853.9	CUU CUU C
33	GAA	941.2	940.9	939.9	GAA GAA UU
34	GAC	917.2	916.9	915.9	C GAC GAC
35	GAG	957.2	956.9	955.9	GAG GAG U
36	GAU	918.2	917.9	916.9	GAU GAU G
37	GCA	917.2	916.9	915.9	GCA GCA UU
38	GCC	893.2	892.9	891.9	GCC GCC G
39	GCG	933.2	933.2	932.2	G GCG GCG
40	GCU	894.2	893.9	892.9	GCU GCU G
41	GGA	957.2	956.9	955.9	GGA GGA U
42	GGC	933.2	933.0	932.0	GGC GGC G

43	GGG	973.2	973.0	972.0	GGG GGG
44	GGU	934.2	933.9	932.9	GGU GGU G
45	GUA	918.2	918.0	917.0	GUA GUA G
46	GUC	894.2	893.9	892.9	GUC GUC G
47	GUG	934.2	933.9	932.9	G GUG GUG
48	GUU	895.2	894.9	893.9	GUU GUU G
49	UAA	902.2	901.9	900.9	UAA UAA U
50	UAC	878.2	877.9	876.9	UAC UAC CC
51	UAG	918.2	917.9	916.9	G UAG UAG
52	UAU	879.2	878.9	877.9	UAU UAU U
53	UCA	878.2	877.9	876.9	UCA UCA U
54	UCC	854.2	853.9	852.9	UCC UCC UU
55	UCG	894.2	893.9	892.9	G UCG UCG
56	UCU	855.1	854.9	853.9	UCU UCU U
57	UGA	918.2	917.9	916.9	UGA UGA C
58	UGC	894.2	893.9	892.9	UGC UGC C
59	UGG	934.2	933.9	932.9	UGG UGG U
60	UGU	895.2	894.9	893.9	UGU UGU C
61	UUA	879.2	878.9	877.9	UUA UUA U
62	UUC	855.1	854.9	853.9	C UUC UUC
63	UUG	895.2	894.9	893.9	G UUG UUG
64	UUU	856.1	855.9	854.9	CC UUU UUU

Table S6 Biolayer interferometry data showing anticodon loops binding to biotin-labelled ssRNA. -1 and +1 indicate the residue before and after the codon of interest. K_D, equilibrium dissociation constant in μM (highlighted with a blue font); k_{off}, dissociation constant in s^{-1}; k_{on}, association constant in $M^{-1}s^{-1}$. M, mean; SD, standard deviation; CV, coefficient of variation; N, number of replicates; /, no binding detected. Bases in red highlight the residues which may form four base pairings. Values of K_D <10 μM were rounded to one decimal place; values of K_D in 10–50 μM were rounded to the nearest 1; values of K_D in 50–150 μM were rounded to the nearest 5; values of K_D in 150–1000 μM were rounded to the nearest 10; values of K_D >1000 μM were rounded to the nearest 100. Conditions: 0–800 μM anticodon loop, 100 mM NaCl, 100 mM $MgCl_2$, 50 mM HEPES, pH 7.2, 20 ºC.

Family Box Codons:

No.	Position					Codon	K_D			k_{off}			k_{on}			N
	-1	1	2	3	+1		M	SD	CV	M	SD	CV	M	SD	CV	
1	G	**A**	**C**	**A**	G	**ACA**	150	39	26%	0.798	0.150	19%	5424	1046	19%	4
1	G	**A**	**C**	**A**	C	**ACA**	160	10	6%	0.456	0.017	4%	2943	282	10%	4
2	C	**A**	**C**	**C**	C	**ACC**	13	2	16%	0.273	0.037	14%	21478	4573	21%	5
2	A	**A**	**C**	**C**	U	**ACC**	40	0.5	1%	0.335	0.007	2%	8366	168	2%	4
3	G	**A**	**C**	**G**	C	**ACG**	110	4.8	4%	0.802	0.064	8%	7327	651	9%	4
3	A	**A**	**C**	**G**	G	**ACG**	145	1.9	1%	0.504	0.009	2%	3491	66	2%	4
4	A	**A**	**C**	**U**	C	**ACU**	530	47	9%	1.283	0.167	13%	2402	268	11%	4
5	U	**C**	**C**	**A**	U	**CCA**	32	2.0	6%	0.203	0.020	10%	6326	262	4%	4
5	A	**C**	**C**	**A**	G	**CCA**	75	12	16%	0.642	0.051	8%	8397	1127	13%	4
6	G	**C**	**C**	**C**	U	**CCC**	12	2.2	18%	0.202	0.021	10%	17103	1742	10%	3
6	A	**C**	**C**	**C**	U	**CCC**	15	5.3	35%	0.298	0.028	9%	20226	5481	27%	5
6	A	**C**	**C**	**C**	G	**CCC**	16	1.4	9%	0.120	0.014	12%	7692	1362	18%	4
7	A	**C**	**C**	**G**	U	**CCG**	65	11	17%	0.421	0.067	16%	6820	1241	18%	4
7	G	**C**	**C**	**G**	U	**CCG**	85	16	19%	0.410	0.095	23%	4884	1488	30%	4
8	C	**C**	**C**	**U**	C	**CCU**	230	26	11%	0.640	0.056	9%	2778	218	8%	4
9	G	**C**	**G**	**A**	C	**CGA**	27	3.1	11%	0.397	0.010	3%	14643	2049	14%	3
9	A	**C**	**G**	**A**	G	**CGA**	45	11.2	25%	0.401	0.053	13%	9968	3022	30%	5
10	C	**C**	**G**	**C**	C	**CGC**	34	2.7	8%	0.553	0.058	10%	16425	2998	18%	4
10	C	**C**	**G**	**C**	C	**CGC**	84	13	15%	0.668	0.032	5%	8046	1085	13%	4
10	C	**C**	**G**	**C**	C	**CGC**	160	19	12%	0.504	0.071	14%	3150	223	7%	5
11	A	**C**	**G**	**G**	C	**CGG**	80	11	14%	1.059	0.110	10%	12987	841	6%	3
11	A	**C**	**G**	**G**	G	**CGG**	90	19	21%	0.973	0.147	15%	10774	909	8%	4
12	C	**C**	**G**	**U**	C	**CGU**	75	18	23%	0.466	0.131	28%	6364	538	8%	4
13	G	**C**	**U**	**A**	C	**CUA**	/	/	/	/	/	/	/	/	/	/
14	C	**C**	**U**	**C**	C	**CUC**	65	5.8	9%	0.322	0.028	9%	4861	179	4%	4
15	A	**C**	**U**	**G**	U	**CUG**	730	216	30%	0.644	0.088	14%	937	214	23%	5
16	G	**C**	**U**	**U**	C	**CUU**	150	34	23%	0.169	0.010	6%	1177	228	19%	4
17	A	**G**	**C**	**A**	C	**GCA**	95	19	20%	0.513	0.108	21%	5353	89	2%	3
18	A	**G**	**C**	**C**	C	**GCC**	15	2.4	16%	0.312	0.037	12%	21595	2269	11%	4
18	C	**G**	**C**	**C**	C	**GCC**	15	1.1	7%	0.134	0.016	12%	9418	1461	16%	4
18	G	**G**	**C**	**C**	G	**GCC**	22	1.7	8%	0.131	0.026	20%	5914	779	13%	3
19	A	**G**	**C**	**G**	A	**GCG**	22	4.0	19%	0.033	0.013	38%	1500	343	23%	3
19	A	**G**	**C**	**G**	U	**GCG**	38	0.8	2%	0.425	0.012	3%	11208	219	2%	3
20	G	**G**	**C**	**U**	C	**GCU**	120	11	9%	0.747	0.004	1%	6507	633	10%	3
21	A	**G**	**G**	**A**	C	**GGA**	120	4.1	3%	0.556	0.034	6%	4755	226	5%	3
21	A	**G**	**G**	**A**	U	**GGA**	140	15	10%	0.785	0.100	13%	5731	1267	22%	3
22	A	**G**	**G**	**C**	C	**GGC**	50	5.7	11%	0.427	0.018	4%	8551	1106	13%	3
22	G	**G**	**G**	**C**	U	**GGC**	50	12	23%	0.136	0.013	10%	2713	327	12%	3
22	G	**G**	**G**	**C**	U	**GGC**	65	8.7	13%	0.092	0.028	30%	1367	219	16%	3
22	C	**G**	**G**	**C**	C	**GGC**	75	3.1	4%	0.714	0.025	3%	9663	394	4%	4
23	A	**G**	**G**	**G**	C	**GGG**	35	2.5	7%	0.418	0.040	10%	11890	1929	16%	3
24	U	**G**	**G**	**U**	C	**GGU**	34	0.5	2%	0.436	0.018	4%	12993	335	3%	3
24	A	**G**	**G**	**U**	C	**GGU**	105	47	45%	0.804	0.092	11%	8424	2432	29%	4
24	A	**G**	**G**	**U**	C	**GGU**	120	13.1	11%	0.624	0.018	3%	5290	732	14%	5

25	A	**G**	**U**	**A**	U	**GUA**	**400**	78	19%	0.666	0.192	29%	1654	234	14%	4
26	C	**G**	**U**	**C**	C	**GUC**	**5.5**	0.5	9%	0.155	0.018	12%	28057	931	3%	3
26	A	**G**	**U**	**C**	A	**GUC**	**17**	0.6	4%	0.308	0.016	5%	17685	1364	8%	4
26	A	**G**	**U**	**C**	C	**GUC**	**46**	7.2	16%	0.352	0.030	9%	7756	847	11%	5
26	G	**G**	**U**	**C**	C	**GUC**	**65**	2.9	5%	0.491	0.011	2%	7317	430	6%	5
27	A	**G**	**U**	**G**	C	**GUG**	**160**	28	18%	0.900	0.121	13%	5813	1340	23%	4
28	A	**G**	**U**	**U**	U	**GUU**	**470**	132	28%	1.246	0.196	16%	2717	504	19%	3
29	G	**U**	**C**	**A**	C	**UCA**	**280**	60.9	22%	0.117	0.017	15%	435	91	21%	5
30	A	**U**	**C**	**C**	C	**UCC**	**73**	8.9	12%	0.501	0.008	2%	6897	855	12%	4
30	C	**U**	**C**	**C**	G	**UCC**	**75**	7.6	10%	0.470	0.025	5%	6439	391	6%	4
30	G	**U**	**C**	**C**	C	**UCC**	**77**	24	32%	0.768	0.213	28%	10124	723	7%	4
30	C	**U**	**C**	**C**	C	**UCC**	**80**	1.8	2%	0.567	0.009	2%	7025	196	3%	4
31	A	**U**	**C**	**G**	C	**UCG**	**310**	31.8	10%	0.272	0.015	6%	903	147	16%	4
32	G	**U**	**C**	**U**	U	**UCU**	**260**	71	27%	0.152	0.036	24%	606	213	35%	4

Table S6 continued. Split-box codons:

No.	Position					Codon	K_D			k_{off}			k_{on}			N
	-1	1	2	3	+1		M	SD	CV	M	SD	CV	M	SD	CV	
1	G	**A**	**A**	**A**	C	AAA	/	/	/	/	/	/	/	/	/	/
2	C	**A**	**A**	**C**	U	AAC	**860**	212	25%	1.315	0.062	5%	1596	409	26%	3
3	G	**A**	**A**	**G**	G	AAG	**1400**	311	22%	1.713	0.457	27%	1248	334	27%	3
4	A	**A**	**A**	**U**	U	AAU	/	/	/	/	/	/	/	/	/	/
5	A	**A**	**G**	**A**	C	AGA	**1000**	203	20%	1.635	0.196	12%	1591	181	11%	3
6	C	**A**	**G**	**C**	G	AGC	**115**	9.8	9%	0.483	0.039	8%	4214	288	7%	4
6	A	**A**	**G**	**C**	C	AGC	**250**	32	13%	0.528	0.077	15%	2109	143	7%	3
7	A	**A**	**G**	**G**	C	AGG	**85**	19	22%	0.603	0.081	13%	7361	1046	14%	3
8	G	**A**	**G**	**U**	U	AGU	**170**	33	20%	1.379	0.158	11%	8004	803	10%	3
9	G	**A**	**U**	**A**	G	AUA	**420**	44	10%	1.559	0.173	11%	3706	99	3%	3
10	G	**A**	**U**	**C**	G	AUC	**1000**	240	24%	1.224	0.139	11%	1226	261	21%	4
11	G	**A**	**U**	**G**	G	AUG	**330**	32	10%	0.810	0.078	10%	2461	24	1%	3
12	A	**A**	**U**	**U**	A	AUU	/	/	/	/	/	/	/	/	/	/
13	G	**C**	**A**	**A**	C	CAA	/	/	/	/	/	/	/	/	/	/
14	G	**C**	**A**	**C**	G	CAC	**230**	50	22%	0.315	0.040	13%	1453	411	28%	3
15	C	**C**	**A**	**G**	C	CAG	**190**	14	7%	0.244	0.049	20%	1303	352	27%	4
15	C	**C**	**A**	**G**	C	CAG	**351**	95	27%	0.403	0.091	23%	1161	151	13%	3
16	C	**C**	**A**	**U**	C	CAU	**450**	73	16%	0.041	0.006	14%	92	17	19%	5
16	C	**C**	**A**	**U**	U	CAU	**570**	33	6%	0.095	0.004	5%	168	7.8	5%	3
17	C	**G**	**A**	**A**	C	GAA	**750**	82	11%	1.038	0.251	24%	1387	304	22%	3
17	C	**G**	**A**	**A**	U	GAA	**810**	160	20%	0.933	0.072	8%	1169	151	13%	3
18	A	**G**	**A**	**C**	G	GAC	**170**	10	6%	0.410	0.040	10%	2443	99	4%	3
18	A	**G**	**A**	**C**	G	GAC	**240**	27	11%	0.668	0.008	1%	2793	331	12%	4
18	G	**G**	**A**	**C**	G	GAC	**250**	35	14%	0.658	0.077	12%	2474	400	16%	5
18	C	**G**	**A**	**C**	G	GAC	**270**	37	14%	0.505	0.079	16%	1898	32	2%	3
19	A	**G**	**A**	**G**	U	GAG	**140**	6.9	5%	0.736	0.082	11%	5139	322	6%	3
20	A	**G**	**A**	**U**	G	GAU	**190**	32	17%	0.761	0.094	12%	3934	357	9%	3
20	G	**G**	**A**	**U**	C	GAU	**190**	35	18%	0.231	0.034	15%	1244	99	8%	3
21	A	**U**	**A**	**A**	G	UAA	/	/	/	/	/	/	/	/	/	/
22	U	**U**	**A**	**C**	C	UAC	**1500**	188	13%	0.332	0.100	30%	230	65	28%	4
23	A	**U**	**A**	**G**	C	UAG	**210**	47	23%	0.424	0.110	26%	2060	60	3%	3
24	G	**U**	**A**	**U**	C	UAU	/	/	/	/	/	/	/	/	/	/
25	A	**U**	**G**	**A**	U	UGA	/	/	/	/	/	/	/	/	/	/
26	G	**U**	**G**	**C**	U	UGC	/	/	/	/	/	/	/	/	/	/
27	A	**U**	**G**	**G**	U	UGG	**370**	6	2%	0.473	0.002	0%	1263	27	2%	3
28	C	**U**	**G**	**U**	U	UGU	/	/	/	/	/	/	/	/	/	/
29	G	**U**	**U**	**A**	C	UUA	/	/	/	/	/	/	/	/	/	/
30	C	**U**	**U**	**C**	C	UUC	**470**	33	7%	0.659	0.009	1%	1405	124	9%	3
31	G	**U**	**U**	**G**	C	UUG	**240**	88	37%	0.292	0.092	31%	1245	90	7%	3
32	G	**U**	**U**	**U**	C	UUU	/	/	/	/	/	/	/	/	/	/

Table S7 Isothermal titration calorimetry raw data showing anticodon loops binding to ssRNA containing a single RNA triplet (light green) or two identical contiguous RNA triplets (dark green). K_D, equilibrium dissociation constant in μM; R, the mole ratio of the loop bound to ssRNA; dH, enthalpy change in kcal/mol; dG, free Gibbs energy change in kcal/mol. Bases in red highlight the residues which may form four base pairing. Conditions: 40 μM ssRNA in cell, 800 μM anticodon loop in syringe, 100 mM NaCl, 100 mM $MgCl_2$, 50 mM HEPES, pH 7.2, 10 ºC.

Codon	Rep	Cell	Syringe	R	K_D μM	dH kcal/mol	dG kcal/mol
**AAA**	1	AAA	GUCGC CU UUU AA GCGAC	/	/	/	/
	2	AAA	GUCGC CU UUU AA GCGAC	/	/	/	/
	1	AAA AAA C	GUCGC CU UUU AA GCGAC	/	/	/	/
	2	AAA AAA C	GUCGC CU UUU AA GCGAC	/	/	/	/
**AAC**	1	AAC	GUCGC CU GUU AA GCGAC	/	/	/	/
	2	AAC	GUCGC CU GUU AA GCGAC	/	/	/	/
	1	AAC AAC C	GUCGC CU GUU AA GCGAC	1.37	8.47	-19.60	-6.57
	2	AAC AAC C	GUCGC CU GUU AA GCGAC	1.31	9.38	-20.10	-6.52
**AAG**	1	AAG	GUCGC CU CUU AA GCGAC	/	/	/	/
	2	AAG	GUCGC CU CUU AA GCGAC	/	/	/	/
	1	AAG AAG U	GUCGC CU CUU AA GCGAC	1.20	11.50	-22.10	-6.40
	2	AAG AAG U	GUCGC CU CUU AA GCGAC	1.21	11.80	-22.00	-6.39
**AAU**	1	AAU	GUCGC CU AUU AA GCGAC	/	/	/	/
	2	AAU	GUCGC CU AUU AA GCGAC	/	/	/	/
	1	AAU AAU U	GUCGC CU AUU AA GCGAC	/	/	/	/
	2	AAU AAU U	GUCGC CU AUU AA GCGAC	/	/	/	/
**UAA**	1	UAA	GUCGC CU UUA AA GCGAC	/	/	/	/
	2	UAA	GUCGC CU UUA AA GCGAC	/	/	/	/
	1	UAA UAA U	GUCGC CU UUA AA GCGAC	/	/	/	/
	2	UAA UAA U	GUCGC CU UUA AA GCGAC	/	/	/	/
**UAC**	1	UAC	GUCGC CU GUA AA GCGAC	/	/	/	/
	2	UAC	GUCGC CU GUA AA GCGAC	/	/	/	/
	1	UAC UAC CC	GUCGC CU GUA AA GCGAC	/	/	/	/
	2	UAC UAC CC	GUCGC CU GUA AA GCGAC	/	/	/	/
**UAG**	1	UAG	GUCGC CU CUA AA GCGAC	/	/	/	/
	2	UAG	GUCGC CU CUA AA GCGAC	/	/	/	/
	1	G UAG UAG	GUCGC CU CUA AA GCGAC	/	/	/	/
	2	G UAG UAG	GUCGC CU CUA AA GCGAC	/	/	/	/
**UAU**	1	UAU	GUCGC CU AUA AA GCGAC	/	/	/	/
	2	UAU	GUCGC CU AUA AA GCGAC	/	/	/	/
	1	AU UAU UAU	GUCGC CU AUA AA GCGAC	/	/	/	/
	2	AU UAU UAU	GUCGC CU AUA AA GCGAC	/	/	/	/
**AGA**	1	AGA	GUCGC CU UCU AA GCGAC	/	/	/	/
	2	AGA	GUCGC CU UCU AA GCGAC	/	/	/	/
	1	AGA AGA U	GUCGC CU UCU AA GCGAC	1.48	15.30	-14.90	-6.25
	2	AGA AGA U	GUCGC CU UCU AA GCGAC	1.54	15.70	-15.00	-6.23
**AGC**	1	AGC	GUCGC CU GCU AA GCGAC	/	/	/	/
	2	AGC	GUCGC CU GCU AA GCGAC	/	/	/	/
	1	C AGC AGC	GUCGC CU GCU AA GCGAC	1.76	3.37	-7.46	-7.10
	2	C AGC AGC	GUCGC CU GCU AA GCGAC	1.82	3.55	-8.13	-7.06
**AGG**	1	AGG	GUCGC CU CCU AA GCGAC	1.05	5.89	-21.30	-6.78
	2	AGG	GUCGC CU CCU AA GCGAC	1.12	4.73	-19.80	-6.91
	1	AGG AGG U	GUCGC CU CCU AA GCGAC	0.63	2.66	-37.20	-7.22
	2	AGG AGG U	GUCGC CU CCU AA GCGAC	0.62	1.93	-32.80	-7.41
**AGU**	1	AGU	GUCGC CU ACU AA GCGAC	/	/	/	/
	2	AGU	GUCGC CU ACU AA GCGAC	/	/	/	/
	1	AGU AGU U	GUCGC CU ACU AA GCGAC	1.17	6.78	-21.10	-6.70
	2	AGU AGU U	GUCGC CU ACU AA GCGAC	1.33	6.92	-21.30	-6.69
**AUA**	1	AUA	GUCGC CU UAU AA GCGAC	/	/	/	/

	2	AUA	GUCGC CU UAU AA GCGAC	/	/	/	/
	1	AUA AUA U	GUCGC CU UAU AA GCGAC	/	/	/	/
	2	AUA AUA U	GUCGC CU UAU AA GCGAC	/	/	/	/
**AUC**	1	AUC	GUCGC CU GAU AA GCGAC	/	/	/	/
	2	AUC	GUCGC CU GAU AA GCGAC	/	/	/	/
	1	C AUC AUC	GUCGC CU GAU AA GCGAC	1.15	8.55	-16.10	-6.57
	2	C AUC AUC	GUCGC CU GAU AA GCGAC	1.34	8.89	-17.70	-6.55
**AUG**	1	AUG	GUCGC CU CAU AA GCGAC	/	/	/	/
	2	AUG	GUCGC CU CAU AA GCGAC	/	/	/	/
	1	G AUG AUG	GUCGC CU CAU AA GCGAC	1.30	18.70	-14.70	-6.13
	2	G AUG AUG	GUCGC CU CAU AA GCGAC	1.10	16.90	-14.20	-6.19
**AUU**	1	AUU	GUCGC CU AAU AA GCGAC	/	/	/	/
	2	AUU	GUCGC CU AAU AA GCGAC	/	/	/	/
	1	AUU AUU AU	GUCGC CU AAU AA GCGAC	/	/	/	/
	2	AUU AUU AU	GUCGC CU AAU AA GCGAC	/	/	/	/
**CAA**	1	CAA	GUCGC CU UUG AA GCGAC	/	/	/	/
	2	CAA	GUCGC CU UUG AA GCGAC	/	/	/	/
	1	CAA CAA UU	GUCGC CU UUG AA GCGAC	/	/	/	/
	2	CAA CAA UU	GUCGC CU UUG AA GCGAC	/	/	/	/
**CAC**	1	CAC	GUCGC CU GUG AA GCGAC	/	/	/	/
	2	CAC	GUCGC CU GUG AA GCGAC	/	/	/	/
	1	CAC CAC C	GUCGC CU GUG AA GCGAC	/	/	/	/
	2	CAC CAC C	GUCGC CU GUG AA GCGAC	/	/	/	/
**CAG**	1	CAG	GUCGC CU CUG AA GCGAC	/	/	/	/
	2	CAG	GUCGC CU CUG AA GCGAC	/	/	/	/
	1	CAG CAG C	GUCGC CU CUG AA GCGAC	/	/	/	/
	2	CAG CAG C	GUCGC CU CUG AA GCGAC	/	/	/	/
**CAU**	1	CAU	GUCGC CU UAG AA GCGAC	/	/	/	/
	2	CAU	GUCGC CU UAG AA GCGAC	/	/	/	/
	1	CAU CAU U	GUCGC CU UAG AA GCGAC	/	/	/	/
	2	CAU CAU U	GUCGC CU UAG AA GCGAC	/	/	/	/
**GAA**	1	GAA	GUCGC CU UUC AA GCGAC	/	/	/	/
	2	GAA	GUCGC CU UUC AA GCGAC	/	/	/	/
	1	GAA GAA CU	GUCGC CU UUC AA GCGAC	1.69	14.00	-12.40	-6.30
	2	GAA GAA CU	GUCGC CU UUC AA GCGAC	1.70	13.10	-11.10	-6.33
**GAC**	1	GAC	GUCGC CU GUC AA GCGAC	/	/	/	/
	2	GAC	GUCGC CU GUC AA GCGAC	/	/	/	/
	1	C GAC GAC	GUCGC CU GUC AA GCGAC	/	/	/	/
	2	C GAC GAC	GUCGC CU GUC AA GCGAC	/	/	/	/
**GAG**	1	GAG	GUCGC CU CUC AA GCGAC	1.26	6.90	-18.00	-6.69
	2	GAG	GUCGC CU CUC AA GCGAC	1.36	7.41	-17.60	-6.65
	1	GAG GAG U	GUCGC CU CUC AA GCGAC	0.73	1.15	-33.90	-7.70
	2	GAG GAG U	GUCGC CU CUC AA GCGAC	0.79	0.55	-27.60	-8.12
**GAU**	1	GAU	GUCGC CU AUC AA GCGAC	/	/	/	/
	2	GAU	GUCGC CU AUC AA GCGAC	/	/	/	/
	1	GAU GAU G	GUCGC CU AUC AA GCGAC	1.13	12.50	-24.20	-6.36
	2	GAU GAU G	GUCGC CU AUC AA GCGAC	1.18	11.90	-23.60	-6.39
**UGA**	1	UGA	GUCGC CU UCA AA GCGAC	/	/	/	/
	2	UGA	GUCGC CU UCA AA GCGAC	/	/	/	/
	1	UGA UGA C	GUCGC CU UCA AA GCGAC	/	/	/	/
	2	UGA UGA C	GUCGC CU UCA AA GCGAC	/	/	/	/
**UGC**	1	UGC	GUCGC CU GCA AA GCGAC	/	/	/	/
	2	UGC	GUCGC CU GCA AA GCGAC	/	/	/	/
	1	UGC UGC C	GUCGC CU GCA AA GCGAC	/	/	/	/
	2	UGG UGG U	GUCGC CU GCA AA GCGAC	/	/	/	/
**UGG**	1	UGG	GUCGC CU CCA AA GCGAC	/	/	/	/
	2	UGG	GUCGC CU CCA AA GCGAC	/	/	/	/
	1	UGG UGG U	GUCGC CU CCA AA GCGAC	/	/	/	/
	2	UGG UGG U	GUCGC CU CCA AA GCGAC	/	/	/	/
**UGU**	1	UGU	GUCGC CU ACA AA GCGAC	/	/	/	/
	2	UGU	GUCGC CU ACA AA GCGAC	/	/	/	/

	1	UGU UGU C	GUCGC CU ACA AA GCGAC	/	/	/	/
	2	UGU UGU C	GUCGC CU ACA AA GCGAC	/	/	/	/
**UUA**	1	UUA	GUCGC CU UAA AA GCGAC	/	/	/	/
	2	UUA	GUCGC CU UAA AA GCGAC	/	/	/	/
	1	A UUA UUA U	GUCGC CU UAA AA GCGAC	/	/	/	/
	2	A UUA UUA U	GUCGC CU UAA AA GCGAC	/	/	/	/
**UUC**	1	UUC	GUCGC CU GAA AA GCGAC	/	/	/	/
	2	UUC	GUCGC CU GAA AA GCGAC	/	/	/	/
	1	C UUC UUC	GUCGC CU GAA AA GCGAC	/	/	/	/
	2	C UUC UUC	GUCGC CU GAA AA GCGAC	/	/	/	/
**UUG**	1	UUG	GUCGC CU CAA AA GCGAC	/	/	/	/
	2	UUG	GUCGC CU CAA AA GCGAC	/	/	/	/
	1	G UUG UUG	GUCGC CU CAA AA GCGAC	/	/	/	/
	2	G UUG UUG	GUCGC CU CAA AA GCGAC	/	/	/	/
**UUU**	1	UUU	GUCGC CU AAA AA GCGAC	/	/	/	/
	2	UUU	GUCGC CU AAA AA GCGAC	/	/	/	/
	1	CC UUU UUU	GUCGC CU AAA AA GCGAC	/	/	/	/
	2	CC UUU UUU	GUCGC CU AAA AA GCGAC	/	/	/	/
**ACA**	1	ACA	GUCGC CU UGU AA GCGAC	/	/	/	/
	2	ACA	GUCGC CU UGU AA GCGAC	/	/	/	/
	1	ACA ACA U	GUCGC CU UGU AA GCGAC	2.18	18.90	-6.61	-6.12
	2	ACA ACA U	GUCGC CU UGU AA GCGAC	2.28	18.40	-6.65	-6.14
**ACC**	1	ACC	GUCGC CU GGU AA GCGAC	1.40	5.72	-13.70	-6.80
	2	ACC	GUCGC CU GGU AA GCGAC	1.36	5.71	-13.90	-6.80
	1	C ACC ACC	GUCGC CU GGU AA GCGAC	2.01	3.78	-19.00	-7.03
	2	C ACC ACC	GUCGC CU GGU AA GCGAC	1.68	4.76	-18.40	-6.90
**ACG**	1	ACG	GUCGC CU CGU AA GCGAC	1.61	10.60	-12.10	-6.45
	2	ACG	GUCGC CU CGU AA GCGAC	1.53	12.40	-10.50	-6.36
	1	ACG ACG	GUCGC CU CGU AA GCGAC	0.92	4.35	-20.60	-6.95
	2	ACG ACG	GUCGC CU CGU AA GCGAC	1.27	7.74	-19.10	-6.63
**ACU**	1	ACU	GUCGC CU AGU AA GCGAC	/	/	/	/
	2	ACU	GUCGC CU AGU AA GCGAC	/	/	/	/
	1	ACU ACU U	GUCGC CU AGU AA GCGAC	2.22	13.20	-11.60	-6.32
	2	ACU ACU U	GUCGC CU AGU AA GCGAC	1.80	12.80	-11.10	-6.34
**CGA**	1	CGA	GUCGC CU UCG AA GCGAC	/	/	/	/
	2	CGA	GUCGC CU UCG AA GCGAC	/	/	/	/
	1	CGA CGA C	GUCGC CU UCG AA GCGAC	/	/	/	/
	2	CGA CGA C	GUCGC CU UCG AA GCGAC	/	/	/	/
**CGC**	1	CGC	GUCGC CU GCG AA GCGAC	/	/	/	/
	2	CGC	GUCGC CU GCG AA GCGAC	/	/	/	/
	1	CGC CGC C	GUCGC CU GCG AA GCGAC	/	/	/	/
	2	CGC CGC C	GUCGC CU GCG AA GCGAC	/	/	/	/
**CGG**	1	CGG	GUCGC CU CCG AA GCGAC	1.98	13.30	-7.62	-6.32
	2	CGG	GUCGC CU CCG AA GCGAC	1.68	14.90	-8.83	-6.25
	1	CGG CGG	GUCGC CU CCG AA GCGAC	2.51	7.03	-5.36	-6.69
	2	CGG CGG	GUCGC CU CCG AA GCGAC	2.45	6.22	-5.28	-6.75
**CGU**	1	CGU	GUCGC CU ACG AA GCGAC	/	/	/	/
	2	CGU	GUCGC CU ACG AA GCGAC	/	/	/	/
	1	CCU CGU UU	GUCGC CU ACG AA GCGAC	1.68	11.90	-12.00	-6.38
	2	CCU CGU UU	GUCGC CU ACG AA GCGAC	1.72	11.50	-11.80	-6.40
	1	CGU CGU UU	GUCGC CU ACG AA GCGAC	2.31	11.90	-5.18	-6.38
	2	CGU CGU UU	GUCGC CU ACG AA GCGAC	2.50	10.90	-5.14	-6.43
**CUA**	1	CUA	GUCGC CU UAG AA GCGAC	/	/	/	/
	2	CUA	GUCGC CU UAG AA GCGAC	/	/	/	/
	1	CUA CUA C	GUCGC CU UAG AA GCGAC	/	/	/	/
	2	CUA CUA C	GUCGC CU UAG AA GCGAC	/	/	/	/
**CUC**	1	CUC	GUCGC CU GAG AA GCGAC	/	/	/	/
	2	CUC	GUCGC CU GAG AA GCGAC	/	/	/	/
	1	CUC CUC CC	GUCGC CU GAG AA GCGAC	1.60	14.8	-18.70	-6.26
	2	CUC CUC CC	GUCGC CU GAG AA GCGAC	1.47	16.2	-19.10	-6.21
**CUG**	1	CUG	GUCGC CU CAG AA GCGAC	/	/	/	/

	2	CUG	GUCGC CU CAG AA GCGAC	/	/	/	/
	1	G CUG CUG	GUCGC CU CAG AA GCGAC	/	/	/	/
	2	G CUG CUG	GUCGC CU CAG AA GCGAC	/	/	/	/
**CUU**	1	CUU	GUCGC CU AAG AA GCGAC	/	/	/	/
	2	CUU	GUCGC CU AAG AA GCGAC	/	/	/	/
	1	C CUU UUUU	GUCGC CU AAG AA GCGAC	/	/	/	/
	2	C CUU UUUU	GUCGC CU AAG AA GCGAC	/	/	/	/
	1	CUU CUU U	GUCGC CU AAG AA GCGAC	2.20	23.60	-10.20	-6.00
	2	CUU CUU U	GUCGC CU AAG AA GCGAC	2.25	20.90	-9.54	-6.06
**GGA**	1	GGA	GUCGC CU UCC AA GCGAC	1.12	5.19	-19.50	-6.85
	2	GGA	GUCGC CU UCC AA GCGAC	1.16	5.42	-19.30	-6.82
	1	GGA U	GUCGC CU UCC AA GCGAC	1.01	3.93	-21.50	-7.01
	2	GGA U	GUCGC CU UCC AA GCGAC	1.02	3.74	-21.90	-7.04
	1	GGA GGA U	GUCGC CU UCC AA GCGAC	0.17	0.62	-31.30	-8.05
	2	GGA GGA U	GUCGC CU UCC AA GCGAC	0.16	0.58	-28.70	-8.10
**GGC**	1	GGC	GUCGC CU GCC AA GCGAC	1.71	1.88	-9.87	-7.42
	2	GGC	GUCGC CU GCC AA GCGAC	1.42	1.08	-7.81	-7.73
	1	GGC GGC G	GUCGC CU GCC AA GCGAC	1.26	2.05	-3.77	-7.65
	2	GGC GGC G	GUCGC CU GCC AA GCGAC	1.36	2.08	-5.25	-7.61
**GGG**	1	GGG	GUCGC CU CCC AA GCGAC	0.32	0.34	-29.40	-8.38
	2	GGG	GUCGC CU CCC AA GCGAC	0.33	0.27	-27.30	-8.51
	1	GGG GGG	GUCGC CU CCC AA GCGAC	/	/	/	/
	2	GGG GGG	GUCGC CU CCC AA GCGAC	/	/	/	/
**GGU**	1	GGU	GUCGC CU ACC AA GCGAC	0.93	4.23	-22.40	-6.97
	2	GGU	GUCGC CU ACC AA GCGAC	1.00	4.93	-22.00	-6.89
	1	GGU GGU G	GUCGC CU ACC AA GCGAC	0.04	5.42	-80.00	-6.83
	2	GGU GGU G	GUCGC CU ACC AA GCGAC	0.04	5.31	-80.00	-6.84
**GUA**	1	GUA	GUCGC CU UAC AA GCGAC	/	/	/	/
	2	GUA	GUCGC CU UAC AA GCGAC	/	/	/	/
	1	GUA GUA G	GUCGC CU UAC AA GCGAC	0.98	14.10	-28.40	-6.29
	2	GUA GUA G	GUCGC CU UAC AA GCGAC	0.80	16.30	-23.60	-6.20
**GUC**	1	GUC	GUCGC CU GAC AA GCGAC	1.54	5.55	-15.60	-6.81
	2	GUC	GUCGC CU GAC AA GCGAC	1.59	5.52	-15.50	-6.82
	1	GUC GUC G	GUCGC CU GAC AA GCGAC	2.16	1.41	-14.10	-7.59
	2	GUC GUC G	GUCGC CU GAC AA GCGAC	1.86	1.03	-15.60	-7.77
**GUG**	1	GUG	GUCGC CU CAC AA GCGAC	1.21	9.46	-14.00	-6.51
	2	GUG	GUCGC CU CAC AA GCGAC	1.30	9.95	-13.80	-6.49
	1	G GUG GUG	GUCGC CU CAC AA GCGAC	0.04	3.84	-68.10	-7.03
	2	G GUG GUG	GUCGC CU CAC AA GCGAC	0.04	3.75	-63.30	-7.04
**GUU**	1	GUU	GUCGC CU AAC AA GCGAC	/	/	/	/
	2	GUU	GUCGC CU AAC AA GCGAC	/	/	/	/
	1	GUU GUU G	GUCGC CU AAC AA GCGAC	0.64	7.44	-36.30	-6.65
	2	GUU GUU G	GUCGC CU AAC AA GCGAC	0.62	6.89	-39.40	-6.69
**UCA**	1	UCA	GUCGC CU UGA AA GCGAC	/	/	/	/
	2	UCA	GUCGC CU UGA AA GCGAC	/	/	/	/
	1	UCA UCA U	GUCGC CU UGA AA GCGAC	/	/	/	/
	2	UCA UCA U	GUCGC CU UGA AA GCGAC	/	/	/	/
**UCC**	1	UCC	GUCGC CU GGA AA GCGAC	2.60	8.55	-4.54	-6.57
	2	UCC	GUCGC CU GGA AA GCGAC	2.52	9.05	-4.76	-6.54
	1	UCC UCC UU	GUCGC CU GGA AA GCGAC	1.24	8.50	-20.40	-6.59
	2	UCC UCC UU	GUCGC CU GGA AA GCGAC	1.30	10.10	-21.90	-6.48
**UCG**	1	UCG	GUCGC CU CGA AA GCGAC	/	/	/	/
	2	UCG	GUCGC CU CGA AA GCGAC	/	/	/	/
	1	CC UCG UUU	GUCGC CU CGA AA GCGAC	/	/	/	/
	2	CC UCG UUU	GUCGC CU CGA AA GCGAC	/	/	/	/
	1	G UCG UCG	GUCGC CU CGA AA GCGAC	/	/	/	/
	2	G UCG UCG	GUCGC CU CGA AA GCGAC	/	/	/	/
**UCU**	1	UCU	GUCGC CU AGA AA GCGAC	/	/	/	/
	2	UCU	GUCGC CU AGA AA GCGAC	/	/	/	/
	1	UCU UCU U	GUCGC CU AGA AA GCGAC	/	/	/	/
	2	UCU UCU U	GUCGC CU AGA AA GCGAC	/	/	/	/

**CCA**	1	CCA	GUCGC CU UGG AA GCGAC	/	/	/	/
	2	CCA	GUCGC CU UGG AA GCGAC	/	/	/	/
	1	CCA UUUUU	GUCGC CU UGG AA GCGAC	1.46	18.30	-10.60	-6.14
	2	CCA UUUUU	GUCGC CU UGG AA GCGAC	1.46	18.10	-10.50	-6.15
	1	CCA CCA UU	GUCGC CU UGG AA GCGAC	1.95	22.60	-13.50	-6.02
	2	CCA CCA UU	GUCGC CU UGG AA GCGAC	1.91	22.40	-13.30	-6.03
**CCC**	1	CCC	GUCGC CU GGG AA GCGAC	1.28	2.77	-20.60	-7.21
	2	CCC	GUCGC CU GGG AA GCGAC	1.28	2.92	-20.90	-7.18
	1	CCC CCC	GUCGC CU GGG AA GCGAC	1.88	0.33	-24.50	-8.41
	2	CCC CCC	GUCGC CU GGG AA GCGAC	1.91	0.55	-25.40	-8.12
	1	CCCCCCCC	GUCGC CU GGG AA GCGAC	3.01	2.44	-24.20	-7.27
	2	CCCCCCCC	GUCGC CU GGG AA GCGAC	3.06	2.49	-24.80	-7.26
**CCG**	1	CCG	GUCGC CU CGG AA GCGAC	2.18	6.91	-6.03	-6.69
	2	CCG	GUCGC CU CGG AA GCGAC	1.95	7.66	-6.23	-6.63
	1	G CCG CCG	GUCGC CU CGG AA GCGAC	/	/	/	/
	2	G CCG CCG	GUCGC CU CGG AA GCGAC	/	/	/	/
**CCU**	1	CCU	GUCGG CU AGG AA CCGAC	/	/	/	/
	2	CCU	GUCGG CU AGG AA CCGAC	/	/	/	/
	1	CCU U	GUCGG CU AGG AA CCGAC	1.41	19.50	-9.52	-6.11
	2	CCU U	GUCGG CU AGG AA CCGAC	1.41	18.00	-9.36	-6.15
	1	CCU CCU UU	GUCGG CU AGG AA CCGAC	1.13	9.68	-22.90	-6.50
	2	CCU CCU UU	GUCGG CU AGG AA CCGAC	1.44	11.40	-19.60	-6.41
**GCA**	1	GCA	GUCGG CU UGC AA CCGAC	/	/	/	/
	2	GCA	GUCGG CU UGC AA CCGAC	/	/	/	/
	1	GCA GCA UU	GUCGG CU UGC AA CCGAC	/	/	/	/
	2	GCA GCA UU	GUCGG CU UGC AA CCGAC	/	/	/	/
**GCC**	1	GCC	GUCGC CU GGC AA GCGAC	1.42	0.66	-15.80	-8.02
	2	GCC	GUCGC CU GGC AA GCGAC	1.50	0.67	-15.40	-8.01
	1	GCC GCC C	GUCGC CU GGC AA GCGAC	2.76	0.42	-12.50	-8.27
	2	GCC GCC C	GUCGC CU GGC AA GCGAC	2.80	0.37	-12.60	-8.35
**GCG**	1	GCG	GUCGC CU CGC AA GCGAC	1.04	1.23	-14.30	-7.66
	2	GCG	GUCGC CU CGC AA GCGAC	0.89	0.95	-15.10	-7.81
	1	G GCG GCG	GUCGC CU CGC AA GCGAC	1.05	0.42	-9.39	-8.27
	2	G GCG GCG	GUCGC CU CGC AA GCGAC	1.21	0.48	-9.25	-8.20
**GCU**	1	GCU	GUCGC CU AGC AA GCGAC	1.34	6.76	-16.20	-6.70
	2	GCU	GUCGC CU AGC AA GCGAC	1.27	6.59	-16.40	-6.72
	1	GCU GCU G	GUCGC CU AGC AA GCGAC	1.47	0.16	-24.20	-8.82
	2	GCU GCU G	GUCGC CU AGC AA GCGAC	1.44	0.15	-21.00	-8.87
**Loop-Loop**		GUCGC CU GAC AA GCGAC	GUCGC CU GUU AA GCGAC	0.63	1.28	-21.3	-7.64
		GUCGC CU GUU AA GCGAC	GUCGC CU GAC AA GCGAC	0.86	3.14	-27.6	-7.13
		GUCGC CU GGC AA GCGAC	GUCGC CU GCC AA GCGAC	0.81	0.99	-16.5	-7.78
		GUCGC CU GGU AA GCGAC	GUCGC CU GCC AA GCGAC	1.38	1.73	-13.5	-7.47

Table S8 Isothermal titration calorimetry data for anticodon loops binding to a ssRNA containing a single triplet. K_D, equilibrium dissociation constant in μM; R, the mole ratio of the loop to ssRNA; dH, enthalpy change in kcal/mol. Blocks in blue highlight the family-box (four-fold degenerate) codons. [a] hairpin forms self-dimer/duplex; [b] ssRNA forms G-quadruplex; [c] ssRNA forms self-dimer.

	U			C			A			G			
	**K_D**	**R**	**dH**	**K_D**	**R**	**dH**	**K_D**	**R**	**dH**	**K_D**	**R**	**dH**	
**U**	/			/			/			/			**U**
	/			/			/			/			**C**
	/			/			/[a]			/			**A**
	/			/			/			/			**G**
**C**	/			/			/			/[c]			**U**
	/			2.8	1.3	-21	/			/[c]			**C**
	/			/			/			/[a]			**A**
	/			/			/			14	1.8[a]	-8.2	**G**[a]
**A**	/			/			/			/			**U**
	/			5.7	1.4	-14	/			/			**C**
	/[a]			/			/			/			**A**
	/			12	1.6	-11	/			5.3	1.1	-21	**G**
**G**	/			6.7	1.3	-16	/			4.6	1.0	-22	**U**
	5.5	1.6	-16	0.7	1.5	-16	/			1.5	1.6	-8.8	**C**[a]
	/			/[a]			/			5.3	1.1	-19	**A**
	9.7	1.3	-14	1.1	1.0	-15	7.2	1.3	-18	0.3	0.3[b]	-28	**G**

Table S9 Isothermal titration calorimetry data for anticodon loops binding to a ssRNA containing two contiguous identical triplets. K_D equilibrium dissociation constant in μM; R, the mole ratio of the loop to ssRNA; dH, enthalpy change in kcal/mol. Boxes in blue highlight the family-box (four-fold degenerate) codons. a) hairpin forms self-dimer/duplex; b) ssRNA forms G-quadruplex; c) ssRNA forms self-dimer; d) possible four base pairings.

	U			C			A			G			
	K_D	R	dH	K_D	R	dH	K_D	R	H	K_D	R	dH	
U	/			/			/			/			U[d]
	/			9.3	1.3	-21	/			/[c]			C
	/			/			/[a]			/			A
	/			/			/			/			G
C	22	2.2	-9.9	11	1.3	-21	/			11	2.4	-5.2	U[d]
	15	1.5	-19	0.4	1.9	-25	/			/[c]			C
	/			22	1.9	-13	/			/[a]			A
	/			/			/			6.6	2.5[a]	-5.3	G[a]
A[d]	/			13	2.0	-11	/			6.8	1.2	-21	U[d]
	8.7	1.2	-17	4.3	1.9	-19	8.9	1.3	-20	3.5	1.8	-7.8	C
	/[a]			19	2.2	-6.6	/			16	1.5	-15	A
	18	1.2	-14	6.1	1.1[c]	-20	12	1.2	-22	2.3	0.6[b]	-35	G
G	7.2	0.6	-38	0.2	1.5	-23	12	1.2	-24	5.4	0.04[b]	-80	U[d]
	1.2	2.0	-15	0.4	2.8[c]	-13	/			1.3	2.1	-4.5	C[a]
	15	0.9	-26	/[a]			14	1.7	-12	0.6	0.2[b]	-30	A
	3.8	0.04[b]	-66	0.5	1.1[b]	-9.3	0.9	0.8[b]	-31	/[b]			G

Table S10 Biolayer interferometry data for two different anticodon loops binding to biotin-labeled ssRNA. -1 and +1 indicate the residue before and after the codon of interest. s, single loop binding to the ssRNA; sq, sequential binding, the second binding kinetics after the first codon is saturated; m, mixed two anticodon loops binding to ssRNA 5'-A UUC CAU C. K_D, equilibrium dissociation constant in μM; k_{off}, disociation constant in s^{-1}; k_{on}, association constant in $M^{-1}s^{-1}$. M, mean; SD, standard deviation; CV, coefficient of variation; N, number of replicates. Data in black indicate family box codons; blue indicate split box codons.

Conditions: 200 μM for the first family-box anticodon loop, 0-400 μM for the second anticodon loop or the single loop, 100 mM NaCl, 100 mM $MgCl_2$, 50 mM HEPES, pH 7.2, 20 ºC.

	Position						Aa	K_D			k_{off}			k_{on}			N
	-1	1	2	3	+1	Codon		M	SD	CV	M	SD	CV	M	SD	CV	
s	A	G	C	C	C	GCC	Ala	**15**	2.4	16%	0.312	0.037	12%	21595	2269	11%	4
sq	A	G	C	C	C	GCC		**13**	0.8	6%	0.055	0.005	9%	4154	222	5%	5
s	C	C	U	C	C	CUC	Leu	**66**	5.8	9%	0.322	0.028	9%	4861	179	4%	4
sq	C	C	U	C	C	CUC		**49**	2.8	6%	0.040	0.009	22%	805	152	19%	5
						5'-A GCC CUC C											
s	A	C	C	C	U	CCC	Pro	**16**	5.3	33%	0.298	0.028	9%	20226	5481	27%	5
sq	A	C	C	C	U	CCC		**16**	1.7	11%	0.224	0.018	8%	14368	692	5%	5
s	C	U	C	C	G	UCC	Ser	**73**	7.6	10%	0.470	0.025	5%	6439	391	6%	4
sq	C	U	C	C	G	UCC		**110**	7.9	7%	0.765	0.167	22%	7027	1171	17%	5
						5'-A CCC UCC G											
s	A	A	C	G	G	ACG	Thr	**145**	3.1	2%	0.509	0.012	2%	3550	146	4%	5
sq	A	A	C	G	G	ACG		**135**	11.9	9%	0.17	0.014	9%	1252	199	16%	4
s	G	G	U	C	C	GUC	Val	**67**	2.9	4%	0.491	0.011	2%	7317	430	6%	5
sq	G	G	U	C	C	GUC		**62**	9.2	15%	0.699	0.049	7%	11506	1355	12%	5
						5'-A ACG GUC C											
s	A	C	G	A	G	CGA	Arg	**43**	11.2	26%	0.401	0.053	13%	9968	3022	30%	5
sq	A	C	G	A	G	CGA		**47**	12.4	26%	0.886	0.135	15%	19730	5732	29%	5
s	A	G	G	U	C	GGU	Gly	**120**	13.1	11%	0.624	0.018	3%	5290	732	14%	5
sq	A	G	G	U	C	GGU		**105**	9.1	9%	0.567	0.020	4%	5365	317	6%	5
						5'-A CGA GGU C											
s	A	G	A	C	G	GAC	Asp	**240**	27	11%	0.668	0.008	1%	2793	331	12%	4
sq	A	G	A	C	G	GAC		**580**	51	9%	1.650	0.012	1%	2852	248	9%	4
s	C	G	C	C	C	GCC	Ala	**14**	1.1	8%	0.134	0.016	12%	9418	1461	16%	4
sq	C	G	C	C	C	GCC		**34**	1.0	3%	0.195	0.010	5%	5787	227	4%	5
						5'-A GAC GCC C											
s	A	G	U	C	U	GUC	Val	**80**	0.8	1%	0.806	0.008	1%	10021	169	2%	4
sq	A	G	U	C	U	GUC		**240**	33.6	14%	0.862	0.071	8%	3595	394	11%	4
s	C	U	A	C	C	UAC	Tyr	**3400**	578	17%	0.504	0.076	15%	153	49	32%	5
sq	C	U	A	C	C	UAC		**/**	/	/	/	/	/	/	/	/	/
						5'-A GAC GCC C											
s	C	**U**	**U**	**C**	C	UUC	Phe	**450**	32	7%	0.635	0.047	7%	1404	124	9%	3
s	C	**C**	**A**	**U**	C	CAU	His	**180**	2.8	2%	0.031	0.001	5%	169	7	4%	3
m						mixed		**190**	9.0	5%	0.024	0.001	5%	127	10	8%	5
						5'-A UUC CAU C											

Table 11 Example of (A) a subset of anticodon loops that have some pairs which can bind each other (all ending in C) and (B) an orthogonal subset. A mismatch in the codon:anticodon pair leads to a considerable decrease in K_D (Fig. S17). The codon:anticodon pair do not permit mismatch or wobble (WB) pairing, while loop-loop interaction allows WB pairing (Fig. S12). If the potentially paired anticodons are present in this anticodon set, (indicated as GGU[1], GGC[2], and GCC[3] in the Table A) the set is self-interacting and should be disregarded. See https://github.com/nieseln/Codon-select.git for code.

(A)

Aa	Codon	Anticodon	Possibly kissed anticodons 5'-3'						
		3WC	3WC	2WC + 1 WB			1WC + 2WB		
Ser	**UCC**	GGA	UCC	UCU	UUC		UUU		
Leu	**CUC**	GAG	CUC	CUU	UUC		UUU		
Pro	**CCC**	GGG	CCC	CCU	CUC	UCC	UUC	UCU	CUU
Arg	**CGC**	GCG*	CGC	UGC	CGU		UGU		
Thr	**ACC**	GGU[1]	ACC	ACU	AUC	GCC[3]	AUU	GUC	GCU
Val	**GUC**	GAC	GUC	GUU					
Ala	**GCC**	GGC[2]	GCC[3]	GCU	GUC		GUU		
Gly	**GGC**	GCC[3]	GGC[2]	GGU[1]					

(B)

Aa	Codon	Anticodon	Possibly kissed anticodons 5'-3'						
		3WC	3WC	2WC + 1 WB			1WC + 2WB		
Ser	**UCC**	GGA	UCC	UCU	UUC		UUU		
Leu	**CUC**	GAG	CUC	CUU	UUC		UUU		
Pro	**CCC**	GGG	CCC	CCU	CUC	UCC	UUC	UCU	CUU
Arg	**CGA**	UCG	CGA	UGA	CGG		CGG		
Thr	**ACG**	CGU	ACG	GCG	AUG		GUG		
Val	**GUC**	GAC	GUC	GUU					
Ala	**GCC**	GGC	GCC	GCU	GUC		GUU		
Gly	**GGU**	ACC	GGU						

Table S12 Fourteen orthogonol family-box codon sets from exhaustive search. Codon sets including GGG were excluded to avoid issues due to quadruplex formation. Set 3 was chosen for sequential binding measurement.

	Ser	Leu	Pro	Arg	Thr	Leu	Ala	Gly
1	UCC	CUC	CCC	CGA	ACG	GUC	GCU	GGU
2	UCC	CUC	CCC	CGA	ACG	GUC	GCU	GGC
**3**	**UCC**	**CUC**	**CCC**	**CGA**	**ACG**	**GUC**	**GCC**	**GGU**
4	UCC	CUC	CCC	CGA	ACG	GUC	GCA	GGU
5	UCC	CUC	CCC	CGA	ACG	GUC	GCA	GGC
6	UCC	CUC	CCC	CGA	ACG	GUC	GCG	GGU
7	UCC	CUC	CCC	CGA	ACG	GUC	GCG	GGC
8	UCC	CUC	CCC	CGG	ACG	GUC	GCU	GGU
9	UCC	CUC	CCC	CGG	ACG	GUC	GCU	GGC
10	UCC	CUC	CCC	CGG	ACG	GUC	GCC	GGU
11	UCC	CUC	CCC	CGG	ACG	GUC	GCA	GGU
12	UCC	CUC	CCC	CGG	ACG	GUC	GCA	GGC
13	UCC	CUC	CCC	CGG	ACG	GUC	GCG	GGU
14	UCC	CUC	CCC	CGG	ACG	GUC	GCG	GGC
